# Environmental impacts from European food consumption can be reduced with carbon pricing or a value-added tax reform

**DOI:** 10.1038/s43016-025-01284-y

**Published:** 2026-01-20

**Authors:** Charlotte Plinke, Michael Sureth, Matthias Kalkuhl

**Affiliations:** 1 Potsdam Institute for Climate Impact Research (PIK), Member of the Leibniz Association, Potsdam, Germany; 2 Faculty VI – Planning Building Environment, Technical University Berlin, Berlin, Germany; 3 Faculty of Economics and Social Sciences, University of Potsdam, Potsdam, Germany

**Keywords:** Environmental economics, Economics, Climate-change policy, Environmental impact

## Abstract

Food consumption generates substantial environmental externalities that remain insufficiently addressed by public policies. Here we explore the global environmental footprints induced by food consumption in the European Union (EU27) based on a multi-regional input–output model, and assess the potential of tax policies for mitigation. Using household expenditure data, we estimate country-specific demand systems for food products and link these to the footprints for the policy analysis. We find that removing current VAT reductions on meat products has the potential to decrease food consumption-related greenhouse-gas emissions, water consumption, land use, biodiversity loss, and the nitrogen and phosphorus footprints of EU27 household food consumption by 3.5%–5.7%. A greenhouse-gas emission price of ~€52 per tCO_2_e on all food products leads to equivalent emission reductions with higher associated environmental co-benefits. The mean net welfare costs of the two policies amount to €12–26 per year per household.

## Abstract (web summary)

The environmental impacts of food consumption in the European Union are global. This study estimates the environmental footprints of EU27 household food consumption, simulating the mitigation potential of tax policies and assessing consumption-related welfare costs.

## Main

The global food system generates large externalities that impose pressure on the environment^1,2^ and contribute to the risk of transgressing a number of planetary boundaries^3,4^. Given the current trends in dietary patterns, these pressures and consequent damages are projected to increase substantially in the next few decades^5–7^. Food consumption in the member states of the European Union (EU27) is a major contributor to the global food system’s externalities^8–10^, which are generated both within and outside the EU through global supply chains^11–13^. There is thus an urgent need to mitigate the environmental pressures induced by the dietary choices of EU27 households, with shifts towards more plant-based diets highlighted as being crucial^3,14^. Consumption taxes have emerged as a particularly effective policy intervention to promote the changes in food consumption patterns necessary for the food system to remain within planetary boundaries^15–19^.

Previous research investigating the effectiveness of externality-correcting policy instruments in the food sector has mainly analysed carbon taxes to reduce the greenhouse gas (GHG) emissions of food consumption^19–27^. Some studies have examined the distributional effects of externality pricing, particularly for carbon and nitrogen^28,29^, but the effectiveness as well as costs and benefits of policies addressing climate and non-climate environmental externalities such as biodiversity loss, water consumption and nutrient emissions have generally not received adequate attention^30^. Furthermore, existing studies often lack a comprehensive consideration of complementing and substitution patterns when assessing consumers’ reactions to policy interventions, and often neglect to contrast the benefits with the costs of policy instruments.

This study analyses the environmental footprints of EU27 households’ food consumption, evaluates the potential of consumption tax policies for mitigation, and assesses the consumption-related welfare costs incurred by households. The analysed environmental impacts are directly associated with the food system and quantified to be at increasing or high risk of transgressing planetary boundaries^4^. To simulate the effect of demand-side policies on households’ food consumption patterns and the resulting footprint reductions, we estimate country-specific price elasticities. To mitigate the environmental pressures from EU27 households’ food consumption, we analyse two specific policy options, which differ in precision in the pricing of external effects, flexibility, administrative costs and the associated time frame for implementation, but yield equal GHG emission reductions.

We first analyse the removal of existing value-added tax (VAT) reductions for meat products, in a similar manner to previous studies^28,31,32^. Currently, 22 out of 27 EU member countries apply reduced VAT rates on meat products, despite their adverse environmental impact. We anticipate that a VAT reform faces relatively low administrative barriers given that it aligns with the EU’s declared sustainability objectives^33^ and reflects a minor reform within a well-established system. Second, we analyse a GHG emission price on all food products. This approach aligns with the European Scientific Advisory Board on Climate Change’s recommendation to introduce emissions pricing in the agricultural sector by 2031^34^ and is under consideration by the EU Commission^35^. An externality-specific pricing is favourable from an economic perspective as it induces a more comprehensive adjustment in production and consumption decisions by directly targeting environmental impacts. However, it involves substantial implementation challenges, including measurement and monitoring of emissions along supply chains, and thus requires considerable time and administrative capacity to be implemented.

The two policies represent distinct approaches to addressing environmental externalities from food consumption. The VAT reform exemplifies a pragmatic short-term solution that leverages existing tax infrastructure but offers limited flexibility. In contrast, the GHG emission price demonstrates how precise externality pricing could work in practice, although it will require more sophisticated implementation mechanisms. By examining both options, we provide insights into the trade-offs between administrative feasibility and economic efficiency in environmental policy design. Our analysis demonstrates how diverse policy instruments can be consistently compared across different cost and benefit dimensions by normalizing with respect to one selected environmental impact (that is, GHG emission reductions). This approach allows quantification of the synergies and trade-offs between environmental impacts while accounting for consumer costs and tax revenues. Finally, we contrast the policy-induced welfare costs with the additional tax revenue generated by the policies.

## Results

### Environmental footprints

We compute the currently observed profile of environmental footprints induced by household food consumption in the EU27 based on the multi-regional input−output model EXIOBASE (base year 2019)^36,37^. Countries’ environmental food consumption footprints are defined as the aggregate global environmental impacts induced by the respective country’s final household food consumption per year. These impacts encompass the total amount of a stressor released (for example, GHG emissions) or the amount of resources used (for example, land) in the production of food within a geographical unit per year. We exclude final demand categories other than final consumption expenditures by households.

Environmental satellite accounts provided in EXIOBASE allow us to relate environmental impacts along the entire global supply chain to final household demand. Specifically, the impacts we analyse are GHG emissions (in CO_2_ equivalents (CO_2_e), excluding emissions from land use and land-use change), blue water consumption (that is, consumptive use of ground and surface water flows), nitrogen and phosphorus emissions and 20 land-use stressors (Supplementary Table 3). To quantify biodiversity loss impacts, each land-use stressor is translated into spatially explicit biodiversity loss in terms of the global potentially disappeared fraction (PDF) of species using region-specific characterization factors^9,38^.

**Fig. 1:**
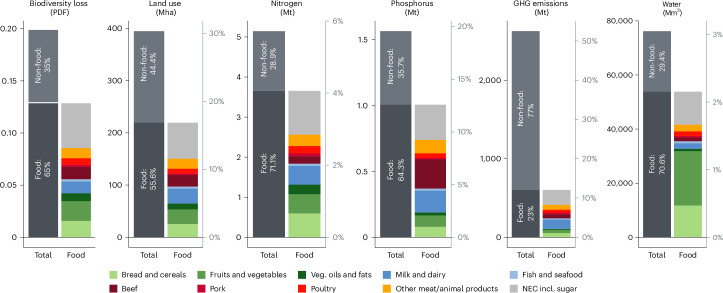
Global environmental footprints resulting from household consumption in the EU27. Breakdown of footprints, distinguishing between food and non-food (left bar), and ten food categories (right bar). The left *y* axes display the absolute footprints in the respective environmental impact measurement unit. The right *y* axes display the share of global food-related planetary boundaries (as quantified by ref. ^3^) attributed to EU27 households’ food consumption. For biodiversity loss, no share can be defined due to the different metrics employed. NEC denotes not elsewhere classified food items. Source data

Figure 1 displays the EU27 households’ global environmental footprints in 2019, categorized into food and non-food consumption, and differentiated into ten food categories. Food consumption accounts for less than a quarter of the total EU27 households’ GHG emission footprint, more than half of the land use, biodiversity loss and phosphorus emission footprints, and more than two-thirds of water consumption and nitrogen emission footprints. In comparison with the global food system’s planetary boundaries^3^, EU27 households require 2.2% (water consumption) to 16.9% (land use) of the global amount that can be safely used for food production (see the secondary *y* axes in Fig. 1), compared to the EU27’s ~5.5% share of the global population.

To investigate the impact of dietary composition, aggregate food consumption is partitioned into ten distinct categories (Supplementary Table 2). The contributions of the different food categories differ substantially by environmental indicator, with meat, other animal products and not elsewhere classified (NEC) food products predominantly influencing all footprints, except for water consumption. Plant-based food categories (‘bread and cereals’, ‘fruits and vegetables’ and ‘veg. oils and fats’) account for 17.8% of GHG emissions, 29.5% of land use, 32.9% of biodiversity loss, and 36% of nitrogen and 18.8% of phosphorus emissions induced by food consumption in the EU. The water footprint is notably higher for plant-based food categories, which account for almost two-thirds of the EU27’s global total water footprint from food consumption.

**Fig. 2:**
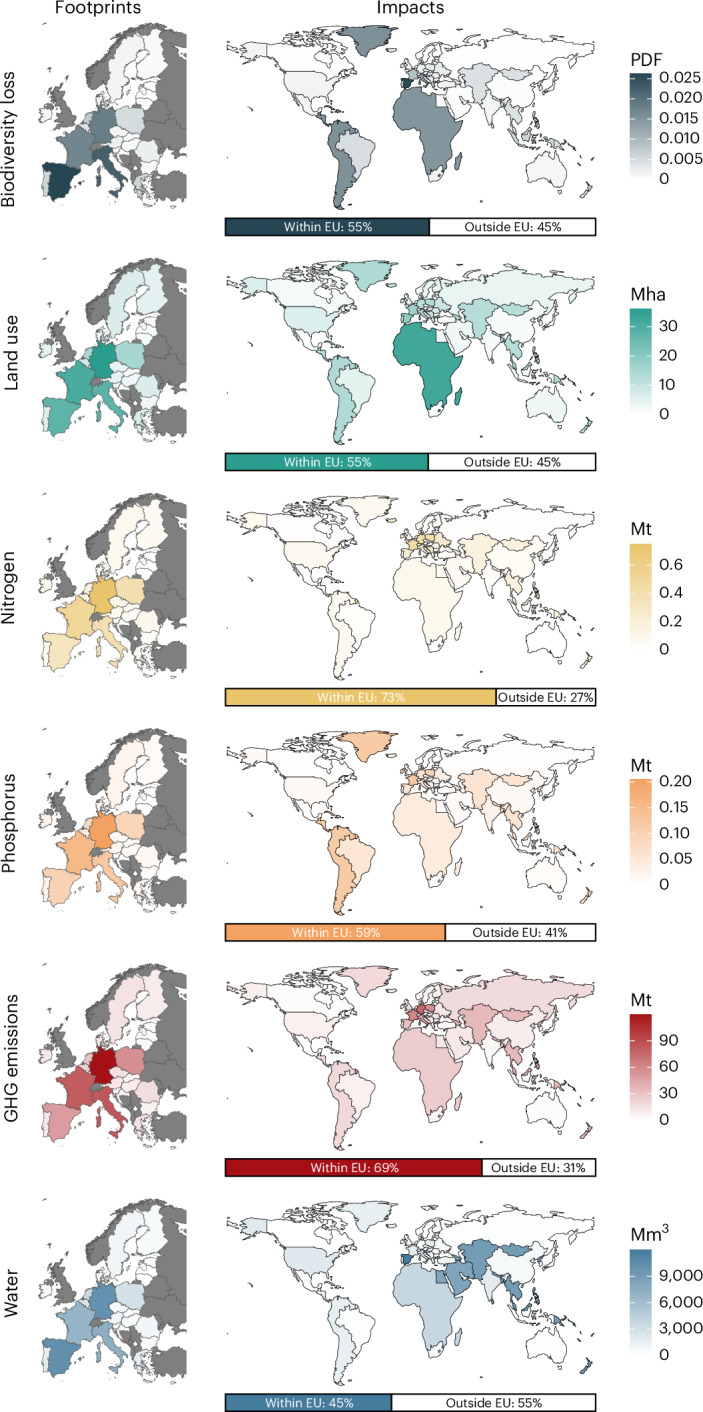
Environmental footprints and impacts of EU27 households’ food consumption. Left: total environmental footprints of households’ food consumption by EU27 country and environmental indicator. Dark grey areas denote countries outside the EU27 (not analysed). Right: spatial allocation of total environmental impacts induced by EU27 households’ food consumption by EXIOBASE region. Bars display the share of impacts occurring within or outside the EU27. Source data

There is substantial variation in environmental footprints among EU countries, as well as the geographic distribution of environmental impacts. The largest food consumption footprints are caused by the most populous countries (Germany, France, Italy, Spain and Poland; Fig. 2, left panels). Per capita environmental footprints by country are presented in Extended Data Table 1. Heterogeneity in the footprints among countries arises not only from variations in dietary composition, but also from environmental impact intensities in the geographic origins of food. Although between 45% and 73% of environmental impacts associated with EU27 food consumption occur within the EU27, a substantial share of impacts are imported from non-EU27 countries (Fig. 2, right panels). This is particularly severe for water consumption (55% imported), for which large shares are imported from countries in Asia (21%) and the Middle East (15%). Biodiversity loss and land use involve the importation of 45% of environmental impacts, with a substantial share originating from countries in Middle and South America and Africa. The majority of phosphorus emissions are caused within EU boundaries (59%), but considerable proportions are also imported from Middle and South America (17%) and Asia (10%). More than two-thirds of GHG emissions and nitrogen emissions originate from countries within the EU, with the largest share of non-EU impacts imported from countries in Asia.

### Policy simulation

We first simulate the removal of VAT reductions on meat products for all countries. This implies that country-specific standard rates instead of reduced VAT rates are applied to the food products falling within the categories *Beef*, *Pork*, *Poultry* and *Other meat/animal products*. We analyse the effect of such a policy on EU27 households’ GHG emissions, land use, biodiversity loss, water consumption, nitrogen and phosphorus footprints.

We next analyse the effects of a CO_2_e-weighted GHG emission price, applied to all food products according to the country-specific mean GHG emission demand intensity of each food category. The CO_2_e-weighted GHG -emission price is set to the level that yields equivalent total GHG emission reductions as the VAT reform. In doing so, the two policies can be compared in terms of their global effects on non-climate environmental impacts and their associated costs to consumers. The resulting endogenously computed GHG emission price leading to equivalent GHG emission reductions as the VAT reform is determined at a level of €51.63 (49.02–53.76) per tCO_2_e.

As a robustness check, we present the respective GHG emission prices under varying demand system estimation specifications in Supplementary Fig. 3, and the associated footprint reductions in Supplementary Fig. 4.

#### Consumer reactions to price changes

**Extended Data Fig. 1:**
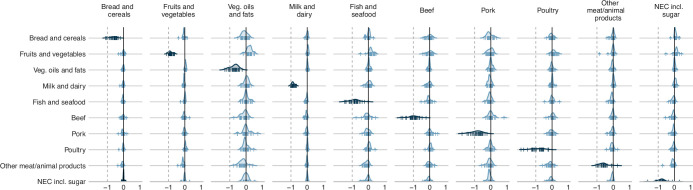
Distribution of mean uncompensated own- and cross-price elasticities across EU27 member states. Each line represents the weighted mean of household-specific own- or cross-price elasticities in one country from the food category given in a specific column to the food categories given in the respective rows. Overlaid on the 27 lines representing the country means is a probability density. Own-price elasticities are highlighted in dark blue. In the analysis, both substitution and income effects are considered such that the full effect of policy-induced price changes on the demanded quantities is captured. We find considerable variation in own- and cross-price elasticities across countries for all food categories. This highlights the importance of estimating country-specific elasticities rather than assuming uniform elasticities for all EU countries. In general, households exhibit relatively lower absolute elasticity in basic food categories such as *Bread and cereals* compared to non-basic food categories like *Beef* or *Pork*. Mean country-specific cross-price elasticities, that is changes in demand for one good in response to a price change of another good, are generally centred around zero with goods acting as complements or substitutes depending on the country-specific household preferences. NEC denotes not elsewhere classified food items. Note: The underlying mean uncompensated own- and cross-price elasticities across EU27 member states are displayed in Supplementary Data 1. Source data

In response to price changes of specific food categories, households may adjust their overall food consumption patterns considering complementary and substitute relationships between food items. These patterns are reflected by own- and cross-price elasticities of demand, which may differ substantially across countries due to varying preferences and income levels^39^. Because comprehensive and representative estimates for all EU27 countries are not available in the literature, we estimate censored linear-approximated Exact Affine Stone Index (EASI) demand systems^40^ based on high-quality representative national household surveys including the Eurostat Household Budget Survey 2015 and 2010, the Konsumerhebung 14/15 (provided by Statistics Austria) and Einkommens- und Verbrauchsstichprobe (EVS) 2018 (provided by the German Federal Statistical Office). In accordance with the classification introduced for the footprint analysis, the surveyed food item expenditures are aggregated into ten food categories (Supplementary Table 2). Extended Data Fig. 1 presents the distribution, across all EU27 countries, of the weighted means of household-specific uncompensated own- and cross-price elasticities.

**Fig. 3:**
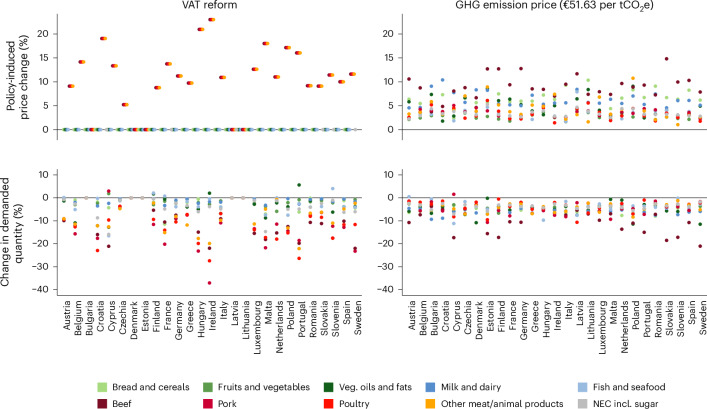
Policy-induced relative price changes and associated demand reactions. Simulated relative changes in prices and resulting demanded quantity changes by food category and country in response to removing VAT reductions for meat products (VAT reform) and implementing a uniform GHG emission price of €51.63 per tCO_2_e on all food products. NEC denotes not elsewhere classified food items. Source data

Figure 3 presents the policy-induced price changes in both policy simulation scenarios and corresponding changes in the demanded quantities. We assume a complete pass-through of the price increase to consumers. The left panels present the policy-induced price changes and accompanying demand changes of a VAT reform (that is, the removal of reduced VAT rates for meat products). For those five countries that do not apply a reduced VAT rate on meat products, the VAT reform does not lead to a change in prices. Removing VAT reductions across the EU27 implies an average price increase for meat products of 10.6%, which reduces demand for the respective meat food groups by 8.0%–11.3%, on average, across all EU27 countries. Although non-meat products are not affected by price increases in this scenario, complementary and substitute relationships between food categories lead to heterogeneous demand effects across countries. In some countries, households substitute meat with fish and seafood, fruits and vegetables or vegetable oils and fats. However, complementary relationships and income effects, that is, a decrease in purchasing power resulting from price increases in meat products, lead to reduced consumption of non-meat food categories in several countries.

The right panels in Fig. 3 present the policy-induced price changes and accompanying demand changes of an EU-wide uniform GHG emission price on all food products, endogenously determined at a price level of €51.63 per tCO_2_e. Although the GHG emission price is uniform, it translates into varying relative price increases across countries and food categories, ranging from 1.0% to 14.8%, contingent on the GHG emission intensities. The GHG emission-intensive food category ‘beef’ is especially affected by large price increases of 9.1%, on average. A GHG emission price imposed on all food categories reduces overall food demand with few exceptions in some countries and categories. In particular, demand for beef decreases by 10.2%, on average, across the EU27, whereas demand reductions for the remaining meat products with lower GHG emission intensities are considerably smaller. In two countries, substitution effects induce an increase in demand for ‘pork’ and ‘fish and seafood’, respectively. Given that in this scenario the GHG emission price is also imposed on non-meat foods, demand also decreases for these products, leading to an overall reduction in food demand in terms of quantities.

#### Footprint reductions

**Fig. 4:**
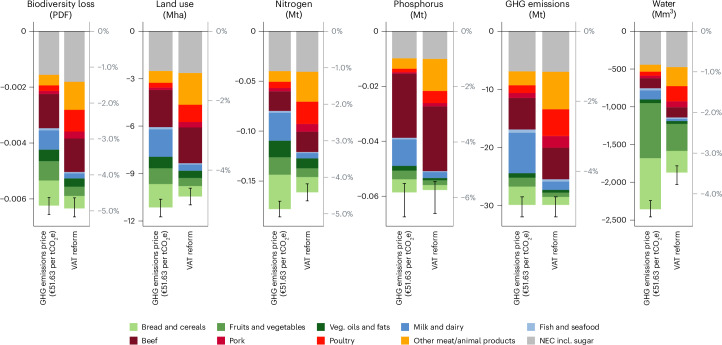
Policy-induced reduction in environmental footprints. Reductions in environmental footprints of EU27 households by food category resulting from removing VAT reductions for meat products (VAT reform) and implementing a GHG emission price of €51.63 per tCO_2_e on all food products. The left *y* axes display the absolute footprint reductions in the respective environmental impact measurement unit. Relative reductions in comparison to current environmental footprints (Fig. 1) are displayed on the right *y* axes. Uncertainty bars represent the range between the minimum and maximum values of the *b* = 100 computed footprint reductions based on the *b* = 100 bootstrapped elasticity estimates. Source data

The changes in consumption patterns induced by the VAT reform translate into global impact reductions of 4.96% (29.9 MtCO_2_e) in terms of GHG emissions, 5.73% (0.058 Mt (megatons)) in terms of phosphorus, 4.41% (0.161 Mt) in terms of nitrogen, 3.48% (1,871 Mm^3^ (cubic megametres)) in terms of water consumption, 4.76% (10.5 Mha (megahectares)) in terms of land used and 4.93% in terms of biodiversity loss (0.0063 global PDF) (Fig. 4).

A GHG emission price of €51.63 per tCO_2_e achieves equivalent total reductions in GHG emission footprints as the VAT reform. However, the GHG emission price policy yields additional global co-benefits in the form of the following reductions: 16,805 t of nitrogen, 891 t of phosphorus, 486 Mm^3^ of water consumption and 0.687 Mha of land use. Only for biodiversity loss are the associated co-benefits of the VAT reform marginally higher (0.0001 global PDF) than those achieved under the GHG emission price policy.

The different food categories’ contributions to the aggregate EU27 footprint reductions varies. The difference between the footprint reductions induced by the VAT reform, which affects only meat prices, and the GHG emission price, which affects prices of all food categories depending on their GHG emission intensity, is evident in the resulting division of footprint reductions between meat and non-meat categories. For the VAT reform, more than half of all footprint reductions (except for water consumption) are due to reductions in meat consumption. In line with demand changes, footprint reductions induced by GHG emission pricing are distributed more equally across all food categories.

#### Welfare analysis

To analyse the consumption-based welfare effects of the two policies, we use the cost-of-living index based on ref. ^40^, representing the increase in expenditures that would be required for a household to sustain the same standard of living with regard to food consumption. The selected welfare metric only captures changes in food-related consumption expenditures and does not account for other welfare-related dimensions, such as health effects or the benefits accruing to EU households from reduced environmental impacts. An assessment of multiple components of broader social welfare measures, including consumer costs, tax revenues and environmental impacts is provided in Supplementary Fig. 5.

**Fig. 5:**
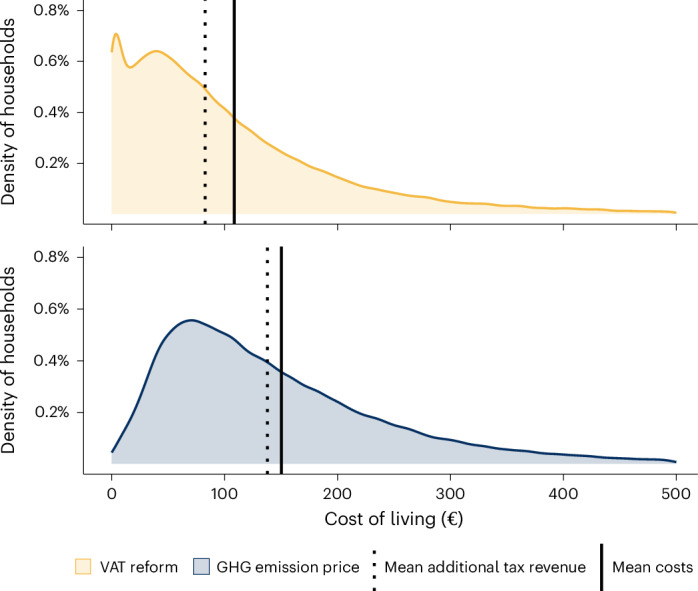
Distribution of welfare costs across all EU27 households. Welfare costs resulting from the removal of VAT reductions for meat products (VAT reform) and a GHG emission price of €51.63 per tCO_2_e on all food products. Household-specific welfare costs are measured by the cost-of-living index (in euros, €) based on the national household survey data. Vertical lines represent the average change in the absolute cost of living (solid line) and the average change in tax income per household (dashed line), comprising the change in VAT income (for both policies) and the additional income from GHG emission pricing (for the GHG emission price policy only). Source data

Figure 5 displays the distribution of policy-induced consumption-based welfare changes across EU27 households. To sustain the same standard of living with regard to food consumption, household food expenditures would, on average, have to increase by €109 due to the VAT reform and by €150 due to a GHG emission price of €51.63 per tCO_2_e (indicated by the solid vertical lines in Fig. 5). The majority of households face higher welfare costs under the GHG emission price policy. This is in line with all food categories being subject to a higher price under this policy, whereas the VAT reform on meat products allows a wider range of substitution choices.

We contrast the increase in food consumption expenditures required to sustain the food-related standard of living with the additional tax revenues from both policies. To do so, we compute the additional available tax revenue per household, incorporating changes in VAT income resulting from both policies, as well as additional income from the GHG emission price. These additional funds could be spent on financial transfers or public goods benefiting households and thus (at least partially) compensate for the policy-induced consumption-based welfare costs. Per household-equivalent, an additional €83 in tax revenue is generated by the VAT reform and an additional €138 in tax revenue by imposing a GHG emission price of €51.63 per tCO_2_e (indicated by the dashed lines in Fig. 5), on average.

The mean consumption-based welfare costs per household exceed the mean additional tax revenue, with an annual difference of €25.81 for the VAT reform policy and €12.43 for the GHG emission price across all EU27 households, corresponding to €11.40 and €5.49 annually per capita, on average. This suggests that both policies may entail relatively modest welfare costs for consumers, provided that tax revenues are effectively redistributed.

Mean estimates of consumption-based welfare costs and additional tax revenues by country are provided in Extended Data Table 2. There is considerable heterogeneity in consumption-based welfare impacts, changes in tax revenue, as well as the resulting net costs to households. In most countries, the gross consumption-based welfare costs are higher for the GHG emission price policy. However, when additional tax revenues can be effectively redistributed, the implementation of a GHG emission price results in lower mean net costs in the majority of countries compared to the VAT reform policy.

## Discussion

This study establishes the link between the environmental footprints of household food consumption in the EU27 and the implementation of policies aimed at mitigating GHG emissions. Previous studies have focused on singular dimensions of food consumption footprints^41–43^ or individual reduction potentials of demand-side policies in the EU^20,22^, but the present study provides an integrated assessment for the EU27 covering a comprehensive range of relevant environmental indicators, as well as changes in tax revenue and consumer costs.

We show that removing the VAT reductions for meat products reduces the environmental footprints induced by the food consumption of EU27 households by 3.5%–5.7%. A GHG emission price on all food products of ~€52 per tCO_2_e achieves equivalent reductions in GHG emissions of 29.9 MtCO_2_e and yields additional global co-benefits in terms of land use, water, nitrogen and phosphorus footprints. Although a GHG emission price alone results in slightly higher costs for consumers than removing VAT reductions, on average, costs to consumers are substantially reduced compared to removing VAT reductions when additional tax revenues are effectively used to compensate consumers.

Our country-specific results suggest differentiated policy priorities based on consumption-based net welfare costs and environmental effectiveness (Extended Data Table 2). Countries with currently high VAT reductions on meat products and relatively high meat consumption, such as Croatia, Hungary and Ireland, could achieve substantial environmental benefits through the VAT reform. For these countries, implementing the VAT reform with targeted compensation measures can improve environmental outcomes in a short time. In contrast, Bulgaria, Denmark and the three Baltic states that already apply standard VAT rates to meat would need to pursue GHG emission pricing directly to reduce emissions—with its longer lead time until policy implementation. However, all EU countries that currently apply reduced VAT rates to meat products except for Czechia, Finland and Slovakia as well as the aggregate of EU countries would achieve lower net costs under a GHG emission price policy compared to the VAT reform, suggesting this instrument would be more favourable in the long run, especially when revenues are used for compensating consumers. This is particularly true in high-income countries such as Belgium, Ireland and Luxembourg, as well as the large economies of Germany and Poland, where the additional tax revenue largely offsets household costs.

We explicitly consider country-specific complementary and substitute relationships between different food categories by estimating demand elasticities for each EU27 country individually. By employing representative country data as well as the most recent demand system methodology, our results demonstrate high accuracy and robustness across various demand system specifications (see ‘Demand system estimation’ and Supplementary Figs. 3 and 4).

In addition to testing the robustness of our results to various assumptions and demand system specifications, we cross-validated our elasticity estimates against available peer-reviewed studies (Supplementary Table 7). We find consistency with previous studies from individual European countries as well as large meta-analyses. Although our elasticity estimates fall within expected ranges, our results demonstrate considerable variation across the EU27 countries, highlighting the importance of using country-specific elasticities rather than uniform values for all countries taken from the literature. The estimated elasticities imply food demand reductions in response to price rises. Our model results may thus imply, under certain conditions, reduced aggregate calorie consumption by households in response to price increases. This relationship conforms to the theoretical basis of demand systems^40^ and is frequently used in studies modelling food demand responses to policy interventions (for example, refs. ^26,44^). It is also in line with the literature finding overall calorie consumption reductions in response to GHG emission tax-induced food price increases^24,45^.

This study has limitations that should be taken into consideration. First, although the administrative implementation of removing VAT reductions on meat products is straightforward, we do not discuss the practical implementation of a GHG emission price policy. It is assumed that the consumer pays the entire additional costs, which depend on the mean country- and food category-specific demand GHG emission intensity, irrespective of the actual origin of the bought item and the point of obligation.

Second, the partial demand system estimation that we conduct considers only households’ food expenditures and allows for unobserved within-category substitution by aggregating products into categories. In particular, by estimating a partial demand system we assume the utility function satisfies weak separability between the commodities represented in the demand system (here, food products) and all other commodities consumed by households. The same weak separability assumption is made for the aggregation of products into categories, where the marginal rate of substitution for products within a category is assumed to be independent of the quantities in other categories^46^. Under these conditions, within-category substitution leads to an optimized (that is, utility-maximizing) bundle of products for each category. Although our estimated elasticities account for potential within-category substitution, the average monetary environmental impact intensities of categories may change in response to the policy interventions due to within-category substitution. Specifically, if the policies lead to an actual intensity reduction due to within-category substitution, we would underestimate, but if they led to an actual intensity increase, we would overestimate the environmental effects of the policies. However, in our analysis, this potential source of bias is not a substantial concern: under the VAT reform scenario, the categories targeted by the policy comprise very homogeneous products, and all products within the categories are subject to the same relative price change. This makes substantial shifts in average emission intensities due to within-category substitution unlikely. In the GHG emission price scenario, within-category substitution to lower-intensity products is incentivized as these products experience lower relative price increases. In this case, post-policy average intensities may be lower than pre-policy, implying that our results can be interpreted as lower-bound estimates on the environmental impact reductions and upper-bound estimates on the costs of the policies (as some margins of adjustments are not considered). The income effect resulting from tax increases may, however, lead in the opposite direction, as it may also imply stronger demand reductions in low-intensity products relative to high-intensity products.

Third, the demand system approach assumes exogenous and constant preferences of consumers, but preferences for low-carbon diets may in fact be endogenous to the policy intervention, for example, in the presence of health benefits^47^.

Fourth, we do not specify the spatial location at which environmental externalities are reduced, given that we cannot determine origin-specific demand responses, but assume demand reductions proportional to current trade and consumption patterns. Reallocations on the producer side are not considered, as the focus of this study is on the global effect of demand-side adjustments. Future research taking into account dynamic trade effects could yield additional insights into the mid- to long-term effects of the investigated policies and shed light on domestic changes in produced impacts rather than global reductions.

Finally, the analysis using EXIOBASE is subject to the caveats of the underlying data. We use data for the year 2019, which rely on ‘now-casting’. Also, the environmental extensions are subject to limitations that warrant emphasis. Our measure of GHG emissions does not include emissions from land use, land-use change and forestry (LULUCF). Due to the challenges in attributing LULUCF emissions to specific sectors, their substantial temporal variability, and the associated measurement uncertainties, we refrain from adding LULUCF emissions from other sources. Given that land-use change can account for up to one-third of GHG emission footprints, in particular for food products imported to the EU^42^, our GHG emission footprints and resulting reductions should be interpreted as conservative estimates. Also, we show that reducing the consumption of products with high environmental intensities has the potential to liberate large land areas and enable new use options. These may have co-benefits regarding GHG emissions and biodiversity, which we do not consider. We also do not explicitly take into account ecosystem deterioration by land-use change, as our measure only captures the equilibrium effect of land occupation. Furthermore, we focus on terrestrial and land use-related biodiversity loss. We do not account for interactions of biodiversity loss with other impacts, such as those related to climate change^48^ or nitrogen emissions^49^. Mitigating these impacts could yield further reductions in biodiversity loss.

Our results imply that the removal of VAT reductions on meat to promote sustainable consumption can be a practicable policy in the short term, as it fosters the reduction of environmental impacts at limited costs for consumers. However, it offers no flexibility for an increase in stringency. In the medium to long term, the reductions achieved by a VAT reform would not be sufficient to meet climate and other environmental objectives. In contrast, a GHG emission price policy allows a gradual increase in its stringency towards the true external GHG emission costs as reflected by the social cost of carbon.

Policymakers aiming to mitigate environmental externalities associated with food consumption should evaluate all relevant welfare dimensions of the different policies. Our framework facilitates this evaluation by normalizing with respect to the primary objective, that is GHG emission reductions. The approach allows for quantifying synergies and trade-offs, and enables a comparison of multiple components of broader social welfare measures, including consumer costs, tax revenues and environmental impacts. By assigning monetary values to changes in environmental footprints, we provide a comprehensive assessment of how each policy affects overall well-being (Supplementary Fig. 5). Our analysis demonstrates that accounting for changes in tax revenue, consumer costs and reductions in domestic nitrogen and phosphorus emissions already results in net-positive aggregate social welfare changes. Accounting for avoided (global) climate damages increases social welfare further.

Larger reductions in food consumption footprints can be expected to be associated with additional costs for consumers compared to the estimates provided in this article. This highlights the importance of implementing revenue redistribution mechanisms, as they have been demonstrated to be pivotal in mitigating the potential regressive effects of policies^28^.

## Methods

### Multi-regional input–output model for environmental footprints

Food consumption by households in the EU is strongly related to environmental impacts outside of the EU boundaries through global supply chains and trade networks. Thus, to determine the complete environmental footprints of EU households’ food consumption and their respective changes due to policy-induced consumption changes, we use a multi-regional input–output (MRIO) model. An input–output model represents the interdependence between different economic sectors by quantifying the flow of goods and services between them.

#### Input–output model

An MRIO model differentiates a standard input–output (IO) model into different regions connected by trade. A standard IO model assumes a single economy of *n* sectors, each producing total output *x*_*i*_.Matrix *A* contains technical coefficients *a*_*ij*_, which represent the input of sector *i* required for sector *j* to produce one unit of output (also referred to as direct requirements). Final demand in each sector *i* is denoted by *y*_*i*_. Using this notation, the economy can be expressed as

1 $${\bf{x}}=A{\bf{x}}+{\bf{y}}$$

This can be reformulated to express total output **x** in terms of final demand **y**:

2 $${\bf{x}}={(I-A)}^{-1}{\bf{y}}$$

To compute the environmental impacts **g** induced by final demand **y** we use the relationship

3 $${\bf{g}}=S{\bf{x}}=S{(I-A)}^{-1}{\bf{y}}$$

where *S* are usage and emission intensities expressed per unit of the economic core.

To differentiate multiple regions, the standard IO model is extended by expanding IO relations to represent the trade between regions. Each region *r* is represented by a standard IO model, where **x**_*r*_ denotes the total output vector, the technical coefficients matrix *A*_*rs*_ contains the inputs required by region *r* from region *s*, and the final demand vector from region *r* to region *s* is denoted by **y**_*rs*_.

#### Data

We use the global MRIO model EXIOBASE (version 3.8.2)^36,37^. The model has a well-established history of use in analysing dietary footprints and modelling dietary shift scenarios^50^. It features global coverage represented by 44 major economies and five rest-of-the-world (RoW) regions. The 27 countries of the EU are represented individually. Furthermore, the model has a detailed and globally consistent sectoral resolution of 200 different product categories. We map the 14 agricultural and ten food-processing sectors onto ten distinct food categories using the concordance table shown in Supplementary Table 2. Of the seven final demand categories included in the model, we only consider final consumption expenditures by households.

EXIOBASE provides detailed satellite accounts containing 1,113 environmental stressors and 126 impact dimensions. This analysis focuses on GHG emissions, nitrogen and phosphorus emissions, freshwater consumption and land-use stressors (Supplementary Table 3). For phosphorus emissions and freshwater consumption, we directly use characterized impacts as provided by EXIOBASE. In addition, we evaluate 20 available land-use stressors, including 13 cropland, three permanent pasture, two forest area stressors, infrastructure, and total other land use. GHG emissions cover six types of GHG emissions (CO_2_, methane (CH_4_), nitrous oxide (N_2_O), sulfur hexafluoride (SF_6_), hydrofluorocarbons (HFCs) and perfluorocarbons (PFCs)), which are converted to CO_2_ equivalents based on most recent estimates of the Global Warming Potential (GWP) 100 metric^51^. Nitrogen emissions, encompassing emissions to water bodies as well as ammonia (NH_3_) and nitrogen oxides (NO_x_) emissions to air, were aggregated into nitrogen impacts using characterization factors provided by EXIOBASE.

We use data for the year 2019. This relies on ‘now-casting’ of the economic structure and all environmental satellite accounts except for CO_2_ emissions. End years of real data points are 2018 for non-CO_2_ emissions and 2011 for all other environmental accounts. As a robustness check, results based on real data points only using data for 2011 are shown in Supplementary Figs. 3 and 4 (specification 3, *2011*).

### Life-cycle impact analysis for biodiversity impacts

To translate the land-use stressors contained in EXIOBASE into spatially explicit impacts in terms of biodiversity loss, we use endpoint characterizations based on LC-IMPACT^52^. The use of spatially explicit characterization factors recognizes that regionalization has been highlighted as crucial to correctly assessing the impact of land use on biodiversity^11^. The resulting measure of biodiversity loss reflects an increase in global extinction risk due to land occupation, but does not explicitly consider land transformation, reflecting the time lag between the pressure and its effects as opposed to an immediate global species loss. The LC-IMPACT model uses the global PDF of species as a proxy for biodiversity loss. The global PDF measures the committed global loss of species richness over time as a direct consequence of anthropogenic impacts on ecosystem quality considering individual species’ vulnerability to deteriorating ecosystem quality given that the pressure (here land occupation) persists^52^. For a single species, a global PDF of 0 reflects no anthropogenic impact, whereas a PDF of 1 corresponds to this species being globally extinct. The global PDF of the LC-IMPACT model is a weighted aggregate of the impact of land use-based stressors on a range of taxonomic groups^52^. As such, the PDF is an average and not a marginal measure of the impact on biodiversity.

Each of the 20 available land-use stressors from EXIOBASE is assigned to one of six land-use types to translate land occupation into region-specific biodiversity loss (Supplementary Table 3)^9,38^. The six land-use types are annual crops, permanent crops, pastures, urban, extensive forestry and intensive forestry. We use region-specific characterization factors for 100-year time horizons consistent with the EXIOBASE mapping determined by ref. ^38^ and made available in an accessible format by ref. ^9^. Using characterization factors *C*, biodiversity loss impacts **g**_BL_ are determined as

4 $${{\bf{g}}}_{{\rm{BL}}}={C}{{\bf{g}}}_{{\rm{LU}}}$$

where **g**_LU_ represents aggregated land-use stressors induced by final demand.

### Demand system estimation

Our analysis requires price elasticities to quantify the effect of price increases on the quantities demanded. There are numerous studies estimating price elasticities for various European countries and food categories^21,23,24,26,32,53^. However, for our purpose, the elasticity estimates in the literature are neither available for all EU27 countries nor consistent with the food categories on which we base our analysis. Thus, we estimate individual demand systems for all EU27 countries to obtain own- and cross-price as well as expenditure elasticities for ten food categories. Specifically, we estimate a linear approximation of the EASI (LA-EASI) implicit Marshallian demand system^40^.

#### Linear approximation of the EASI demand system

The EASI demand system is the most recently developed demand system estimation technique, which has substantial advantages over the previously widely used Almost Ideal Demand System (AIDS^54^) and Quadratic Almost Ideal Demand System (QUAIDS^55^). In general, the EASI demand system offers a more comprehensive and flexible approach to estimating consumer demand compared to AIDS and QUAIDS. Most importantly, EASI demand systems can estimate nonlinear, flexible Engel curves, which is in line with empirical evidence^56^. Furthermore, demands are not constrained by Gorman’s rank restriction, error terms can be interpreted as unobserved preference heterogeneity, and, as it is based on expenditure functions, it allows for the derivation of welfare metrics^40^. We provide a detailed outline of the methodology below and include a summary of all parameter definitions in Supplementary Table 5.

To implement the EASI implicit Marshallian demand system^40^, we assume that household *h* with observable characteristics ${{\bf{z}}}_{h}={({z}_{h,\,1},\,\ldots, \,{z}_{h,\,L})}^{{\prime} }$, unobservable preference characteristics $\bf{{\varepsilon}}_{h}={({\varepsilon }_{h,1},\ldots,{\varepsilon }_{h,n})}^{{{\prime} }}$ with **1**_*n*_**ε**_*h*_ = 0 and log nominal total food expenditures *x*_*h*_ chooses a bundle of *n* food categories facing the vector of log prices **p**_*h*_. Thus, following ref. ^40^, the log food expenditure (or cost) function of household *h* can be specified as *x*_*h*_ = *C*(**p**_*h*_, *u*_*h*_, **z**_*h*_, **ε**_*h*_), expressing the minimum total log expenditures required for household *h* with characteristics **z**_*h*_ and preferences **ε**_*h*_ to realize utility level *u*_*h*_ when facing log prices **p**_*h*_.

Using Shephard’s lemma, the Hicksian budget share functions can be expressed as

5 $${{\bf{w}}}_{h}={\boldsymbol{\omega }}({{\bf{p}}}_{h},{u}_{h},{{\bf{z}}}_{h},{{\bf{\varepsilon}}}_{h})={{\rm{\nabla }}}_{p}C({{\bf{p}}}_{h},{u}_{h},{{\bf{z}}}_{h},{{\bf{\varepsilon}}}_{h})$$

where ∇_*p*_ denotes the vector differential operator evaluated for a change in the price vector **p**. By replacing indirect utility *u*_*h*_ = *V*(**p**_*h*_, *x*_*h*_, **z**_*h*_, **ε**_*h*_) = *C*^−1^(**p**_*h*_, ⋅, **z**_*h*_, **ε**_*h*_) with the implicit utility *y*_*h*_ = *g*(**w**_*h*_, **p**_*h*_, *x*_*h*_, **z**_*h*_), one obtains the so-called implicit Marshallian demand (or budget share) function

6 $${{\bf{w}}}_{h}={\boldsymbol{\omega }}({{\bf{p}}}_{h},{y}_{h},{{\bf{z}}}_{h},{{\bf{\varepsilon}}}_{h})$$

The linear approximation of the EASI demand system assumes that implicit utility *y*_*h*_ = *g*(**w**_*h*_, **p**_*h*_, *x*_*h*_, **z**_*h*_) can be interpreted as the log of real expenditures^40^, which are log nominal expenditures *x*_*h*_ deflated with the Stone price index:

7 $${y}_{h}={x}_{h}-{{\bf{p}}}_{h}^{{\prime} }{{\bf{w}}}_{h}$$

We use observed expenditure shares **w**_*h*_ to exploit the available household-specific information. We acknowledge that this approach introduces endogeneity, but the linear approximation of the EASI demand system using the Stone price index as defined here has been shown to yield estimates that do not differ substantially from those based on the exact system^40^. As a robustness check, results using an alternative EASI specification approximating real expenditures using a common Stone price deflator for all households ${\widetilde{y}}_{h}={x}_{h}-{{\bf{p}}}_{h}^{{\prime} }{\bar{{\bf{w}}}}_{h}$ based on the sample mean of expenditure shares, $\bar{{\bf{w}}}$, are displayed in Supplementary Figs. 3 and 4 (specification 2, *ytilda*). To reduce numerical problems, *y*_*h*_ can be centred in the polynomial regression^40^. As we did not encounter any numerical problems, our main specification uses the uncentred *y*_*h*_. As a robustness check, results based on *y*_*h*_ centred by its sample median, ${y}_{h}^{{\rm{c}}{\rm{e}}{\rm{n}}\mathrm{tr}{\rm{e}}{\rm{d}}}={y}_{h}-\bar{y}$, are displayed in Supplementary Figs. 3 and 4 (specification 4, *ycentered*).

A convenient estimable functional specification of equation (6) is the following system of equations:

8 $${{\bf{w}}}_{h}=\underbrace{\mathop{\sum}\limits_{r=0}^{R=4}{\bf{b}}_{r}{y}_{h}^{r}}_{({\rm{I}})}+\underbrace{{A}{\bf{p}}_{h}}_{({\rm{II}})}+\underbrace{{C}{\bf{z}}_{h}}_{({\rm{II}}{\rm{I}})}+\underbrace{{B}{\bf{p}}_{h}{y}_{h}}_{({\rm{IV}})}+\underbrace{{D}{\bf{z}}_{h}{y}_{h}}_{({\rm{V}})}+{{\mathbf{\epsilon}}}_{{h}}$$

where **w**_*h*_ is an *n*-vector of budget shares that household *h* spends on the *n* food categories. The budget share depends on log real total expenditures *y*_*h*_, **p**_*h*_ is an *n*-vector of the food categories’ log prices faced by household *h*, **z**_*h*_ is the *L*-vector of observable sociodemographics, and **b**, *A*, *B*, *C* and *D* are parameter vectors and matrices to be estimated.

The polynomials in term (I) of equation (8) allow for flexible Engel curves, which describe the relationship between budget shares and total expenditures. Term (II) captures the effects of all *n* prices, and term (III) includes the sociodemographic characteristics that serve as demand shifters, capturing observable heterogeneity between households. The sociodemographics vector **z**_*h*_ contains dummy coded information for age (household head is older than 45 years), gender of the household head, presence of children in the household, household income (household income is above median) and urbanity (household is located in a high-population-density area). The terms (IV) and (V) are interaction terms between expenditures and prices, and expenditures and household characteristics, which capture additional heterogeneity in household responses. As a robustness check, results based on equation (8) amended by an additional interaction term ${\sum }_{l=1}^{L}{z}_{h,\,l}{{E}}_{l}{{\bf{p}}}_{h}$ to account for the interaction between household characteristics and prices is shown in Supplementary Figs. 3 and 4 (specification 7, *pzintyes*).

The model is estimated for each EU27 country individually using sample-weighted seemingly unrelated regression (SUR) to account for the potential correlation of errors across equations within the same household. As a robustness check, results using non-weighted SUR are displayed in Supplementary Figs. 3 and 4 (specification 5, *unweighted*).

Due to data constraints, we estimate partial demand systems resting on the assumption of weak separability between food and non-food consumption^57^. In other words, we assume that households first allocate their budget to general consumption categories such as housing, energy, mobility, food and so on. Then, households choose an optimal mix of commodities within each category given their budget constraint for this category (see also refs. ^23,32,58^). Hence, our demand system estimation only considers food categories and disregards expenditures on other commodities and services. This translates into the assumption that households’ nominal food expenditures remain constant when facing food price changes. As a robustness check, we alternatively estimate incomplete demand systems by including a composite numéraire good that represents all other non-food goods and services consumed by households. Due to a lack of required data for Germany, incomplete demand systems can be estimated for the remaining 26 EU countries only. Results based on the incomplete demand systems thus need to be compared to the partial demand system results excluding Germany in Supplementary Figs. 3 and 4 (specification 6, *incomplete*; specification 9, *partialnoDE*).

#### Adjusting for the censored distribution of budget shares

Due to the restricted duration of recording periods in the household surveys (14 days to 3 months), yearly expenditures for specific food categories may be recorded as zero for some households even though extending the observation period may have resulted in the recording of positive expenditures. Thus, the true expenditure values are only recorded for a subset of all surveyed households. This is a typical sample selection problem where zero observations are the result of an upstream binary choice problem^59,60^. The resulting censored distribution of the dependent variable of the demand system, the vector of budget shares **w**_*h*_, may result in biased and inconsistent parameter estimates. Therefore, the use of procedures that account for the censored distribution is necessary. As this is not accounted for in the original LA-EASI model specification^40^, we apply the widely adopted two-step approach for censored distributions of the dependent variable^61^.

First, we assume a latent variable for the budget share of food category *i*, ${{w}}_{hi}^{* }$, and a sample selection process that governs which budget shares are observed and which are not:

9 $$\begin{array}{l}{{w}}_{{hi}}={d}_{{hi}}{{w}}_{{hi}}^{\ast }\,\,\,{\rm{w}}{\rm{i}}{\rm{t}}{\rm{h}}\,\,\,{d}_{{hi}}=\left\{\begin{array}{l}\begin{array}{cc}1 & \mathrm{if}\,{d}_{hi}^{\ast } > 0\end{array}\\ \begin{array}{cc}0 & \mathrm{if}\,{d}_{hi}^{\ast }\le 0\end{array}\end{array}\right.\end{array}$$

We estimate the probability that the observed budget equals the latent budget share by separately regressing for each food category *i* a binary outcome variable, *d*_*i*_, indicating zero expenditures or not, on a vector of household characteristics **s**_*h*_ using probit models:

10 $${d}_{hi}={{\bf{s}}}_{h}^{{\prime} }{{\boldsymbol{\gamma }}}_{i}+{\zeta }_{hi}$$

The use of separate probit models implies the assumption of Cov(*ζ*_*hi*_, *ζ*_*hj*_) = 0 for *i* ≠ *j* (ref. ^61^). Using the estimated vector ${\widehat{{\boldsymbol{\gamma }}}}_{{\boldsymbol{i}}}$, the standard normal cumulative distribution function (cdf) *Φ*(⋅) and probability density function (pdf) *ϕ*(⋅) are computed:

11 $${\widehat{\varPhi }}_{hi}({{\bf{s}}}_{h}^{{\prime} }{\widehat{{\boldsymbol{\gamma }}}}_{i})\,\,\,{\rm{a}}{\rm{n}}{\rm{d}}\,\,\,{\widehat{\phi }}_{hi}({{\bf{s}}}_{h}^{{\prime} }{\widehat{{\boldsymbol{\gamma }}}}_{i})$$

In the second step, the parameters in equation (8) are estimated correcting for the censored distribution of the observed budget shares (see also ref. ^62^) with the estimated cdf and pdf values:

12 $${{\bf{w}}}_{h}={\hat{\Phi}}_{h}\left[\mathop{\sum }\limits_{r=0}^{R=4}{{\bf{b}}}_{r}{y}_{h}^{r}+{{A{\bf{p}}}}_{h}+{{C{\bf{z}}}}_{h}+{{B{\bf{p}}}}_{h}{y}_{h}+{{D{\bf{z}}}}_{h}{y}_{h}\right]+{\hat{\phi}}_{h}{\bf{f}}+{\mathbf{\epsilon}}_{h}$$

Matrices ${\widehat{\varPhi }}_{h}$ and ${\widehat{\phi }}_{h}$ are *n* × *n* identity matrices where the diagonal elements have been replaced by the estimated cdf and pdf values given by equation (11). Homogeneity is satisfied in equation (12) by the use of (log-)normalized prices. That is, we divide all prices by total food expenditures: ${{\bf{p}}}_{h}=\mathrm{ln}(\frac{{\widetilde{{\bf{p}}}}_{h}}{{x}_{h}})$. The symmetry of the Slutsky matrix is ensured by imposing the symmetry of matrices *A* and *B* as restrictions in the estimation process. The adding-up restriction does not hold in censoring-corrected demand systems in general, and thus cannot be guaranteed by straightforward parametric restrictions^58,63^. We therefore use all *n* equations in the estimation procedure. As a robustness check, results for the uncorrected demand systems, estimated using *n* − 1 equations and imposing the adding-up restriction, are shown in Supplementary Figs. 3 and 4 (specification 1, *uncensored, nmin1*).

#### Prices in the absence of price data

The estimation procedure of the LA-EASI demand system requires the input of prices for each food category included in the demand system. A common problem in demand system estimation is the limited availability of price survey data that can be matched to the household survey data, including food expenditures and quantities^62,64^. Thus, we use information on quantities purchased per food category available in the household survey data to compute unit values. Unit values are defined as food category expenditure per unit purchased. This is a common approach in the absence of price data. As the products within each food category purchased by each household may vary in quality and price across households, we adjust the unit values^65^ (see also, for example, refs. ^32,66^).

Specifically, we regress computed household- and food category-specific unit values UV_*hi*_ on a vector of household characteristics **t**_*h*_. For each of the *n* food categories, the estimated regression model is given by

13 $${\rm{UV}}_{{hi}}={\alpha }_{i}+{{\bf{t}}}_{h}^{{\prime} }{\boldsymbol{\beta }}_{i}+{\xi }_{{hi}}$$

where **t**_*h*_ contains information on the gender and age of the household head as well as the urbanity of the household location, household size, number of children in the household, and household income (except for Italy where data on household income are missing).

We define the *n*-vector of adjusted unit value prices as the sum of the regression constant and the predicted residual values:

14 $${{\bf{p}}}_{h}^{\mathrm{UV}}=\widehat{{\boldsymbol{\alpha }}}+{\widehat{{\boldsymbol{\xi }}}}_{h}$$

By correcting for quality effects, the adjusted prices proxy the variation in unit values due to supply-side factors. As for households with zero expenditures and where for quantities purchased in a given food category no unit values can be computed, we impute the country median of adjusted unit values. Also, in some countries and for some food categories, the number of households that consumed a specific food category may be relatively low, and we estimate equation (13) only if at least 30 household observations with positive expenditures are available. If fewer than 30 households in country *c* consumed food category *i*, no unit value adjustment is conducted, and the non-adjusted unit value is imputed for the adjusted unit value. As a robustness check, results based on non-adjusted unit values are presented in Supplementary Figs. 3 and 4 (specification 8, *uvnonadj*).

#### Elasticities

Price elasticities express the percentage change in quantity demanded of food category *i* due to a 1% price change in food category *j* (called own-price elasticity for *i* = *j* and cross-price elasticity for *i* ≠ *j*). The expenditure elasticities express the percentage change in quantity demanded for food category *i* due to a 1% change in real expenditures *y*.

From the estimated parameters of equation (12), compensated (Hicksian) elasticities can be computed by dividing Hicksian semi-elasticities by the budget share *w*_*hi*_ (ref. ^40^). The Hicksian semi-elasticities with regard to prices are given by

15 $${\nabla}_{p}^{\prime}{\bf{w}}_{h}={\widehat{\Phi}}_{h}[{A}+{B}{y}_{h}]$$

and the Hicksian semi-elasticities with regard to real expenditures are

16 $${\nabla}_{y}{\bf{w}}_{h}={\hat{\Phi}}_{h}\left[\mathop{\sum }\limits_{r=1}^{R=4}r{\bf{b}}_{r}{y}_{h}^{r-1}+{{B{\bf{p}}}}_{h}+{{D{\bf{z}}}}_{h}\right]$$

Thus, own- and cross-price elasticities are computed using

17 $${\eta}_{h}^{\mathrm{PE}}={\mathop{\omega}\limits^{\sim}}_{h}^{-1}{\hat{\Phi}}_{h}[{A}+{B}{y}_{h}]+{\mathop{\omega}\limits^{\sim}}_{h}-I$$

where ${\eta }_{h}^{\mathrm{PE}}$ is an *n* × *n*-matrix of compensated price elasticities of household *h*, ${\widetilde{{\omega }}}_{h}$ is an identity matrix with the ones replaced by the budget shares **w**_*h*_ of household *h*, and *I* is an *n* × *n* identity matrix.

The expenditure elasticities are computed using

18 $${\boldsymbol{\eta}}_{h}^{\mathrm{EE}}={\mathop{\omega}\limits^{ \sim }}_{h}^{-1}{\hat{\Phi }}_{h}\left[\mathop{\sum }\limits_{r=1}^{R=4}r{{\bf{b}}}_{r}{y}_{h}^{r-1}+{{B{\bf{p}}}}_{h}+{{D{\bf{z}}}}_{h}\right]+{{\bf{1}}}_{n}$$

where ${{\boldsymbol{\eta }}}_{h}^{\mathrm{EE}}$ is the *n* × 1-vector of compensated expenditure elasticities of household *h*.

The compensated elasticities can then be transformed to uncompensated elasticities using the Slutsky equation. Uncompensated (Marshallian) elasticities reflect both substitution and income effects and are used in the policy analysis to compute percentage changes in demanded quantities due to price changes.

Based on the estimated demand system parameters we compute household-specific elasticity estimates. To evaluate country-specific changes in demand due to policy-induced price changes, we compute country-specific weighted-mean elasticities. The household-specific elasticities are weighted by household expenditures and sample weights to ensure the representativeness of the country-specific elasticity estimate with regard to overall expenditures and household composition. The resulting country-specific elasticities are consistent with values found in the literature (Supplementary Table 7) and fall within expected ranges. Supplementary Data 1 provides mean uncompensated own- and cross-price elasticities across EU27 member states (elas) and their standard deviations (s.d.).

#### Data

To estimate country-specific demand systems of food consumption, we use the Eurostat Household Budget Survey (HBS) for the reference years 2015 and 2010 for 25 out of 27 EU countries. The national household surveys, which are harmonized in the 2015 (2010) HBS data, were conducted by national statistical offices in the years 2014–2016 (2008–2011) and expenditures in euros are adjusted to the respective HBS reference year using price coefficients^67^. Due to data being missing for Austria and Germany in the HBS dataset, we also use the Konsumerhebung 2014/15 provided by Statistics Austria^68^, as well as the Einkommens- und Verbrauchsstichprobe (EVS) 2018 provided by the German Federal Statistical Office^69^. All 27 surveys are representative cross-sectional sample surveys of private households. A common feature is that they collect households’ expenditure information classified along the UN Classification of Individual Consumption by Purpose (COICOP) using diaries maintained over a fixed time period, which varies between countries from two weeks to 3 months^67^. The data also include selected sociodemographic information of the household and its members (Supplementary Table 4).

For each country covered by the HBS, we consider the most recent available dataset that contains both expenditures and quantities of food items purchased. Specifically, we only use HBS 2010 data if country *c* has no quantity data for at least one food item available per food category (the aggregation of COICOP level 4 food items into ten food categories is detailed in the following). We remove households that report implausible values on the five-digit (level 4) COICOP classified food items; that is, households are excluded if they report (1) negative expenditures or (2) zero expenditures in combination with positive quantities.

Two cases of missing quantity data have to be considered in the HBS datasets. Case 1 is that the quantities consumed of certain food items *i* have not been recorded in country *c* (Supplementary Table 4). Case 2 is that the quantities have been recorded but the recorded data are implausible, as for household *h* the quantity of food item *i* is zero and expenditures for food item *i* are greater than zero. Given the absence of a standardized procedure in the existing literature, we address both cases of missing quantity data separately as outlined in the following.

To address case 1, we use a cross-country matching and imputation algorithm. First, we construct four regions (north, east, south, west) based on the UN geo-scheme for Europe. For each country *c*, we impute quantities for all food items that have not been recorded. To do so, we construct a matching pool containing all households with positive expenditures from all countries that (1) belong to the same region *r* as country *c* and (2) for which quantity data for food item *i* have been recorded. For each household *h* of country *c* that has positive expenditures, the ten nearest neighbours in the matching pool with regard to selected sociodemographic variables, income and expenditures on the specific food item *i* are found within the matching pool. The missing quantity of food item *i* for household *h* in country *c* is then imputed with the mean of the ten nearest neighbours, weighted by their inverted distance and scaled with the ratio of the distance-weighted-mean expenditures of the ten neighbours and household *h*’s expenditures. For households with zero expenditures, zero quantities are imputed.

To address case 2, we use a within-country matching and imputation algorithm. For each country *c*, we impute quantities for all household food item observations that have zero quantities but positive expenditures recorded. To do so, we construct a matching pool containing all households from country *c* with positive expenditures and positive quantities for food item *i*. For each household *h* that has positive expenditures but a zero quantity recorded for food item *i*, the ten nearest neighbours with regard to selected sociodemographic variables, income and expenditures on the specific food item *i* are found within the matching pool. The missing quantity for food item *i* of household *h* is then imputed with the mean of the ten nearest neighbours, weighted by their inverted distance and scaled with the ratio of the distance-weighted mean expenditures of the ten nearest neighbours and household *h*’s expenditures.

We aggregate the five-digit (level 4) COICOP classified expenditures on food items into ten distinct food categories (Supplementary Table 2). In addition to the exclusion criteria mentioned above, we exclude (1) households with unreasonably high food expenditures relative to total expenditures (>75%) and (2) households with zero total food expenditures after the matching procedure. Budget shares of different food categories by country are presented in Supplementary Fig. 1. Using the final data on all EU27 countries, we estimate 27 separate LA-EASI demand systems for each country to obtain country-specific elasticity estimates for the ten food categories as described above.

### Policy simulation

We simulate two policies inducing different price changes across food categories. For the VAT reform policy, we compute relative price changes of the meat categories using country-specific information on reduced and standard VAT rates (Supplementary Table 1):

19 $$\frac{\Delta {p}_{c,\,{\rm{m}}{\rm{e}}{\rm{a}}{\rm{t}}}}{{p}_{c,\,{\rm{m}}{\rm{e}}{\rm{a}}{\rm{t}}}}=\frac{1+{r}_{c,\,{\rm{s}}{\rm{t}}{\rm{a}}{\rm{n}}{\rm{d}}{\rm{a}}{\rm{r}}{\rm{d}}}}{1+{r}_{c,\,{\rm{r}}{\rm{e}}{\rm{d}}{\rm{u}}{\rm{c}}{\rm{e}}{\rm{d}}}}-1$$

where *c* denotes countries and *r*_*c*_ are the VAT rates in country *c*. This implies that the relative price increase is the same for all meat categories and zero for all non-meat categories.

For the GHG emission price policy, the percentage price increase is determined by country-specific GHG emission intensities and computed as

20 $$\frac{\Delta {p}_{{ci}}}{{p}_{{ci}}}={\mu }_{{ci}}{\tau }_{{\rm{G}}{\rm{H}}{\rm{G}}}$$

where *μ*_*ci*_ is the country-specific GHG emission intensity of demand for food category *i*, displayed in Supplementary Fig. 2. This implies relative price increases for all food categories that vary across categories and countries. The relative price increase, $\frac{\Delta {p}_{{ci}}}{{p}_{{ci}}}$, only depends on *μ*_*ci*_ as the GHG emission price level, *τ*_GHG_, isuniform across all food categories and countries. However, as GHG emission intensities are expressed in monetary terms (tCO_2_e per euro), relative price increases are influenced by the GHG emission content per physical unit of each food category as well as the pre-policy price per physical unit.

The usage of monetary intensities (kgCO_2_e per euro) is due to the fact that the satellite accounts of EXIOBASE only provide monetary values and no quantities. Just like physical emission intensities (kgCO_2_e kg^−1^), monetary emission intensities can be appropriately used for implementing a Pigouvian tax. The monetary emission intensity, *μ*, of a product can be expressed as

21 $$\mu =\frac{E/Q}{p}=\frac{E/Q}{X/Q}=\frac{E}{X}$$

where *E* are total emissions, *Q* is physical quantity, *p* is price per physical quantity and *X* is total expenditure. This shows that the monetary intensity *μ* (=*E*/*X*) is simply the physical intensity per unit, *E*/*Q*, divided by the price per unit, *p* = *X*/*Q*.

When multiplying the monetary intensity by the product price (where *τ* is the GHG emission price given in euros per tCO_2_e):

22 $$\Delta p=\mu \times \tau \times p=\frac{E}{X}\times \tau \times \frac{X}{Q}=\frac{E}{Q}\times \tau$$

we recover exactly the carbon price applied to physical emissions.

This leads to proper pricing of the externality if the GHG emission price of *τ* (in € tCO_2_e^−1^) ensures that consumers face the full social cost of their consumption choices. For any given product, the tax payment equals monetary intensity times the GHG emission tax times total expenditures:

23 $$\mu \times \tau \times X=\frac{E}{X}\times \tau \times {pQ}=\frac{E}{X}\times \tau \times \frac{X}{Q}Q=\frac{E}{Q}\times \tau \times Q=E\times \tau$$

This equals the product’s emissions multiplied by the GHG emission price and thereby adheres to the principle of Pigouvian taxation. The seemingly counterintuitive result presented above that some lower GHG emission intensity food categories face large relative price increases is due to their low pre-policy price per unit. A GHG emission price that will lead to an absolute €1 price increase for products with the same physical emissions per unit will lead to a larger percentage price increase for a €2 product compared to a €20 product. However, although relative price changes may vary, the absolute carbon price signal in € tCO_2_e^−1^ remains constant across all food categories and ensures that the externality is priced uniformly across categories.

We assume that the additional cost due to the GHG emission price is not subject to additional VAT. To establish the GHG emission price level (*τ*_GHG_) necessary to achieve equivalent emission reductions as the VAT reform, the model is iteratively solved.

The vector of percentage changes in the demanded quantities of all food categories in country *c* depends on both own- and cross-price elasticities:

24 $$\frac{\Delta {{\bf{q}}}_{c}}{{{\bf{q}}}_{c}}={\eta }_{c}^{\mathrm{PE}}\frac{\Delta {{\bf{p}}}_{c}}{{{\bf{p}}}_{c}}$$

where ${\eta }_{c}^{\mathrm{PE}}$ is the uncompensated elasticity matrix computed as the country-specific weighted mean from ${\eta }_{{ch}}^{\mathrm{PE}}$ (equation (17)), and $\frac{\Delta {{\bf{p}}}_{c}}{{{\bf{p}}}_{c}}$ is the vector of relative price changes across all food categories. Thus, the change in footprints associated with consumption of the *n* food categories in country *c* is computed as

25 $$\Delta {{\bf{g}}}_{c}=\frac{\Delta {{\bf{q}}}_{c}}{{{\bf{q}}}_{c}}{{\bf{g}}}_{c}$$

### Welfare analysis

The contrasting policies’ effects on environmental impacts with their corresponding welfare-related costs require a household welfare metric. Given that the EASI demand system is based on an expenditure function model, the estimated parameters of the demand system can be used to compute a closed-form expression of consumer welfare called log cost-of-living (COL)^40^. As we only capture households’ responses to policy-induced price changes in the demand for food, this welfare metric captures the relative increase in expenditures for food required to sustain the same standard of living with regard to food. Mathematically, it is defined as

26 $$\begin{array}{rcl}\log ({{\rm{COL}}}_{h}) & = & C({{\bf{p}}}_{h1},{u}_{h0},{{\bf{z}}}_{h},{\bf{\varepsilon}}_{h})-C({{\bf{p}}}_{h0},{u}_{h0},{{\bf{z}}}_{h},{\bf{\varepsilon}}_{h})\\ & = & ({{\bf{p}}}_{h1}-{{\bf{p}}}_{h0}){{\bf{w}}}_{h0}+\frac{1}{2}{({{\bf{p}}}_{h1}-{{\bf{p}}}_{h0})}^{{\prime}}({A}+{B}{{y}}_{h})({{\bf{p}}}_{h1}-{{\bf{p}}}_{h0})\end{array}$$

where *C*(⋅) is the log expenditure function. The log COL index is equal to the difference in pre-policy log expenditures, *C*(**p**_*h*0_, *u*_*h*0_, **z**_*h*_, **ε**_*h*_), and log expenditures required to achieve the original level of utility **u**_*h*0_ at post-policy prices **p**_*h*1_. Thus, a positive value corresponds to a welfare loss as households need to expend more to achieve the same level of utility. The index comprises both a first-order effect reflecting a change in purchasing power (given by the Stone index for the price change) and a second-order effect capturing substitution effects between food categories. With major substitution in response to price increases, the latter effect will reduce the overall welfare loss. To obtain absolute monetary values in euros that represent the additional expenditures required to sustain the standard of living with regard to food, we multiply the log(COL_*h*_) by households’ total nominal food expenditures, *x*_*h*_:

27 $$\Delta {\rm{C}}{\rm{O}}{{\rm{L}}}_{h}=\left[({\bf{p}}_{h1}-{\bf{p}}_{h0}){\bf{w}}_{h0}+\frac{1}{2}{({\bf{p}}_{h1}-{\bf{p}}_{h0})}^{\prime}({A}+{{By}}_{h})({\bf{p}}_{h1}-{\bf{p}}_{h0})\right]{x}_{h}$$

We contrast the additional household expenditures required to sustain the standard of living with regard to food with the additional mean tax income per household generated by the two policies in each country *c*. For the VAT reform, we determine the change in VAT paid by household *h* ($\Delta {T}_{h}^{VAT}$) as

28 $$\Delta {T}_{h}^{{\rm{V}}{\rm{A}}{\rm{T}}}=\mathop{\sum }\limits_{i}\left({x}_{hi}^{0}\frac{{r}_{ci}^{0}}{1+{r}_{ci}^{0}}-{x}_{hi}^{1}\frac{{r}_{ci}^{1}}{1+{r}_{ci}^{1}}\right)$$

where ${x}_{hi}^{0}$ is the observed pre-policy household food expenditures in food category *i*, ${x}_{hi}^{1}$ is the post-policy household food expenditures, and ${r}_{ci}^{0}$ and ${r}_{ci}^{1}$ are pre- and post-policy VAT rates applied to food category *i* in country *c* in which household *h* resides. Note that for non-meat categories and countries that already apply the standard VAT rate to meat products, ${r}_{ci}^{1}={r}_{ci}^{0}$.

For the GHG emission price policy, we compute the GHG emission price paid by household *h* ($\Delta {T}_{h}^{GHG}$) as

29 $$\Delta {T}_{h}^{{\rm{G}}{\rm{H}}{\rm{G}}}=\mathop{\sum }\limits_{i}\left({x}_{hi}^{1}\frac{1}{1+{r}_{ci}^{0}}{\mu }_{ci}\,{\tau }^{\mathrm{GHG}}\right)$$

where *μ*_*ci*_ is the mean GHG emission demand intensity in € tCO_2_e^−1^ applied to the net expenditures (${x}_{hi}^{1}\frac{1}{1+{r}_{ci}^{0}}$) in food category *i* for country *c* in which household *h* resides and *τ*^GHG^ is the EU-wide GHG emission price level applied. As the GHG emission price policy will also shift demand among the food categories and in some countries different VAT rates are applied to different food categories, we also determine the change in VAT paid by household *h* for the GHG emission price policy:

30 $$\Delta {T}_{h}^{{\rm{V}}{\rm{A}}{\rm{T}}}=\mathop{\sum }\limits_{i}\left({x}_{hi}^{0}\frac{{r}_{ci}^{0}}{1+{r}_{ci}^{0}}-{x}_{hi}^{1}\frac{{r}_{ci}^{0}}{1+{r}_{ci}^{0}}\right)$$

As ${x}_{hi}^{1}$ cannot be observed, we derive the vector of post-policy food expenditures from budget share semi-elasticities:

31 $${{\bf{x}}}_{h}^{1}=({\bf{1}}+\Delta {{\bf{p}}}^{{\prime} }{{\rm{\nabla }}}_{p}^{{\prime} }{w}_{h}){{\bf{x}}}_{h}^{0}$$

where $\Delta {{\bf{p}}}^{{\prime} }$ is the vector of price changes and ${{\rm{\nabla }}}_{p}^{{\prime} }{w}_{h}$ is a matrix of Hicksian price semi-elasticities as given by equation (16). Each element of this matrix gives the change in the budget share *w*_*i*_ due to a relative price change in food category *j*, that is, $\frac{{\rm{\partial }}{w}_{{hi}}}{{\rm{\partial }}{p}_{{hj}}}$. In the case of the VAT reform, each vector element *i* of $\Delta {{\bf{p}}}^{{\prime} }$ is $\frac{\Delta {p}_{i}}{{p}_{i}}=\frac{1\,+{r}_{i}^{1}}{1+{r}_{i}^{0}}-1$. In the case of the GHG emission price policy, each vector element *i* of $\Delta {{\bf{p}}}^{{\prime} }$ is $\frac{\Delta {p}_{i}}{{p}_{i}}={\mu }_{{ci}}{\tau }_{{\rm{G}}{\rm{H}}{\rm{G}}}$.

Using the household-specific changes in VAT paid and GHG emission price tax revenue we compute EU averages using household and population weights to ensure representativeness.

### Monetization of environmental benefits

To allow for an overall evaluation of to what extent the policies increase global aggregate well-being, we monetize the changes in environmental footprints using the global social cost of GHGs, the domestic social cost of nitrogen and the domestic social cost of phosphorus. As the changes in environmental footprints cannot be mapped to the location of impact, we assume that reductions occur proportional to where impacts are generated in the status quo. The social costs represent the welfare-equivalent monetary value of the net damage caused by the emission of an additional unit of the respective substance and thus also represent the net benefit to society resulting from the reduction of emissions by one unit.

The social cost of GHGs encompasses CO_2_, CH_4_, N_2_O, HFCs, PFCs and SF_6_. We compute the social benefits from reduced GHG emissions using two different sources. First, we refer to ref. ^70^, which provides social cost estimates for CO_2_, CH_4_ and N_2_O. As specific values for HFCs, PFCs and SF_6_ are not available in ref. ^70^, we convert these gases into their CO_2_ equivalent (CO_2_e) and apply the social cost of CO_2_. We also compute the benefits using ref. ^71^, which provides a substantially higher mean estimate for the social cost of carbon (US$283 versus US$193 per tCO_2_). As ref. ^71^ does not provide social cost values for CH_4_ and N_2_O, we impute those values using the ratios between their social cost and the social cost of CO_2_ derived from ref. ^70^. HFCs, PFCs and SF_6_ are also converted into CO_2_e and valued according to the social cost estimate for CO_2_ in ref. ^71^. The social cost of nitrogen is quantified based on estimates from ref. ^72^, which includes the external costs of damage caused by nitrogen leaching and run-off, as well as ammonia (NH_3_) and nitrogen oxides (NO_x_) emissions. Both damage to human health and ecosystem impacts are accounted for. The social cost of phosphorus emissions is quantified based on ref. ^73^, which estimates the external cost of phosphorus emissions into surface waters to be €153.50 per kg. This value is used to compute the local impacts associated with pollution within all EU27 countries. As no robust social cost of nitrogen and phosphorus estimates are available globally, only the value of benefits accruing within the EU is determined. An overview of the social costs assumed is presented in Supplementary Table 6.

### Uncertainty quantification

As the MRIO model is provided without associated uncertainty ranges, the only source of uncertainty underlying the policy simulation arises from the estimated elasticities. To quantify the range of uncertainty surrounding our main results, we employ a non-parametric bootstrapping procedure (ref. ^74^, p. 438), where, for each country *c*, we treat the original household survey dataset of size *N*_*c*_ as the population and draw *b* random samples of size *N*_*c*_ with replacement. We set *b* = 100. Based on each of the *b* bootstrapping samples, we estimate country-specific demand systems as described in the section ‘Demand system estimation’ to obtain country-specific elasticity estimates. Following the same procedure as described in the section ‘Policy simulation’, we then compute the VAT-equivalent GHG emission price and the footprint reductions for both policy simulations for each of the *b* obtained country-specific elasticities. This yields *b* GHG emission price and footprint reduction values. We report the uncertainty as the range between the minimum and maximum of the *b* computed GHG emission price and footprint reduction values.

### Reporting Summary

Further information on research design is available in the Nature Portfolio Reporting Summary linked to this Article.

## Supplementary information

Supplementary Information Supplementary Tables 1–7 and Figs. 1–5.

Reporting Summary

Supplementary Data 1 Price and expenditure elasticities by country (mean and standard deviations).

## Source data

Source Data Fig. 1 Statistical source data.

Source Data Fig. 2 Statistical source data.

Source Data Fig. 3 Statistical source data.

Source Data Fig. 4 Statistical source data.

Source Data Fig. 5 Statistical source data.

Source Data Extended Data Fig. 1 Statistical source data.

## Figures and Tables

**Extended Data Table 1:**
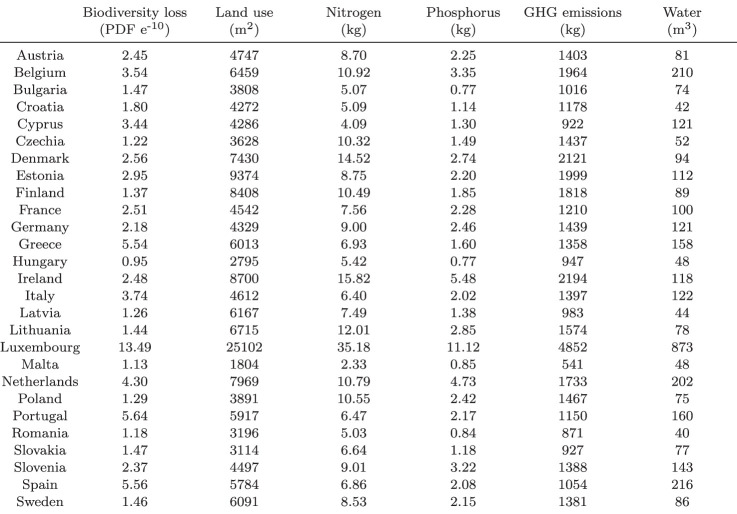
Environmental footprints of households’ food consumption per capita

The table presents environmental footprints associated with households’ food consumption per capita and year by EU27 country. Computations are based on EXIOBASE v.3.8.2 (year 2019).

**Extended Data Table 2:**
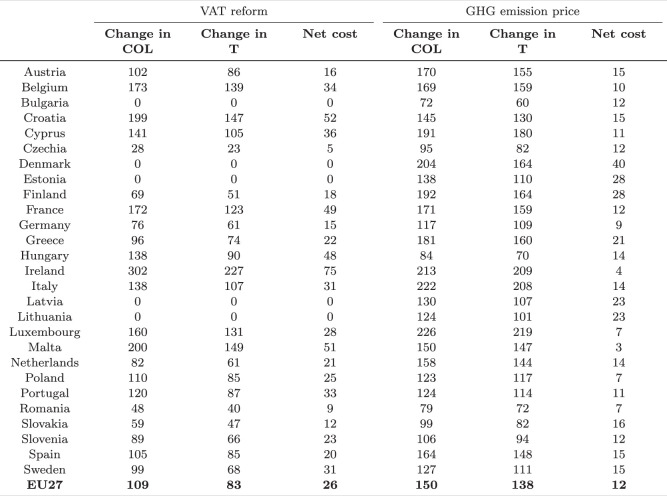
Mean per household welfare impacts of policies in EUR

The table presents welfare effects resulting from the removal of value-added tax reductions for meat products (VAT reform) and the implementation of a GHG emission price of 51.63 EUR/tCO2eq on all food products. It compares the mean change in the absolute cost-of-living (Change in cost) with the mean change in tax income (Change in tax revenue, comprising the change in VAT income and the additional income from GHG emission pricing) by country. Net cost represents the difference between mean change in the absolute cost-of-living and the mean additional tax income. All values are rounded annual means per household in EUR, weighted using sampling weights to ensure representativeness.

## Data Availability

Household microdata were sourced from multiple surveys including Eurostat’s Household Budget Survey (HBS 2010 and 2015, https://ec.europa.eu/eurostat/web/microdata/household-budget-survey), the Konsumerhebung 2014/15 provided by Statistics Austria (https://www.statistik.at/ueber-uns/erhebungen/personen-und-haushaltserhebungen/konsumerhebung) and the Einkommens- und Verbrauchsstichprobe (EVS) 2018 provided by the German Federal Statistical Office (https://www.forschungsdatenzentrum.de/de/10-21242-63231-2018-00-00-3-1-0). Access to data for research purposes must be requested directly from the relevant statistical agencies. Additional statistics on population size and the number and average size of households by country were retrieved from Eurostat’s Population and Demography database (https://ec.europa.eu/eurostat/web/population-demography/demography-population-stock-balance/database) and the Eurostat Labour Force Survey (LFS) database (https://ec.europa.eu/eurostat/web/lfs/database). Environmentally extended multi-regional input–output (MRIO) data were retrieved from EXIOBASE (version 3.8.2) (10.5281/zenodo.5589597)^37^. Land use-related biodiversity loss coefficients were sourced from ref. ^9^ based on ref. ^38^. Social costs of GHGs, nitrogen and phosphorus were taken from refs. ^70–73^. Shapefiles used to create maps are based on Natural Earth Data (https://www.naturalearthdata.com/), were sourced using the rnaturalearth R package (https://cran.r-project.org/web/packages/rnaturalearth/) and in part processed following ref. ^9^. Source data are provided with this paper.

## Code availability

All code to replicate the findings and display items of this study is deposited on Zenodo (10.5281/zenodo.17348643)^75^.
