# Supplementary material for: Environmental impacts from European food consumption can be reduced with carbon pricing or a value-added tax reform
Source: Nat Food. 2026 Jan 20;7(1):74–87. doi: 10.1038/s43016-025-01284-y (PMC12851928; doi:10.1038/s43016-025-01284-y)
Supplement: Supplementary file 1 — Supplementary Tables 1–7 and Figs. 1–5. [file 43016_2025_1284_MOESM1_ESM.pdf]

nature food

Supplementary information https://doi.org/10.1038/s43016-025-01284-y

Environmental impacts from European food consumption can be reduced with carbon pricing or a value-added tax reform

In the format provided by the authors and unedited

Table of contents

Supplementary Table 1: Reduced meat and standard VAT rates by country 2
Supplementary Table 2: Correspondence table of food categories to COICOP and EXIOBASE products 3
Supplementary Table 3: Stressors/Impacts from EXIOBASE (v3.8.2) 4
Supplementary Table 4: Sample characteristics by country 6
Supplementary Table 5: Symbols used in section *Methods – Demand system estimation.* 7
Supplementary Table 6: Social cost estimates 8
Supplementary Table 7: Comparison of own-price elasticities across studies 10
Supplementary Fig. 1: Budget shares of different food categories by country 11
Supplementary Fig. 2: Greenhouse gas emission intensities of demand by food category 12
Supplementary Fig. 3: Robustness of GHG emission price estimates 13
Supplementary Fig. 4: Robustness of environmental footprint reduction estimates 14
Supplementary Fig. 5: Policy-induced monetised social welfare change per household 15

Supplementary Table 1: Reduced meat and standard VAT rates by country.

	Meat VAT (2023)	Standard VAT (2023)
Austria	10.0	20.0
Belgium	6.0	21.0
Bulgaria	20.0	20.0
Croatia	5.0	25.0
Cyprus	5.0	19.0
Czechia	15.0	21.0
Denmark	25.0	25.0
Estonia	20.0	20.0
Finland	14.0	24.0
France	5.5	20.0
Germany	7.0	19.0
Greece	13.0	24.0
Hungary	5.0	27.0
Ireland	0.0	23.0
Italy	10.0	22.0
Latvia	21.0	21.0
Lithuania	21.0	21.0
Luxembourg	3.0	16.0
Malta	0.0	18.0
Netherlands	9.0	21.0
Poland	5.0	23.0
Portugal	6.0	23.0
Romania	9.0	19.0
Slovakia	10.0	20.0
Slovenia	9.5	22.0
Spain	10.0	21.0
Sweden	12.0	25.0

Supplementary Table 2: **Correspondence table of food categories to COICOP and EXIOBASE products.** The table shows the mapping of COICOP (structure level 4/ subclasses (five-digit)) as well as EXIOBASE agricultural and food processing sectors included in the analysis (24 out of 200) and their assignment to the corresponding food category. NEC denotes not elsewhere classified food items.

Category	COICOP	EXIOBASE
Bread and cereals	01111, 01112, 01113, 01114, 01115, 01116, 01117, 01118	'Paddy rice', 'Wheat', 'Cereal grains nec', 'Crops nec', 'Processed rice'
Fruits and vegetables	01161, 01162, 01163, 01164, 01165, 01166, 01167, 01168, 01169, 01171, 01172, 01173, 01174, 01175, 01176, 01177, 01178	'Vegetables, fruit, nuts'
Veg. oils and fats	01152, 01153, 01154	'Oil seeds', 'products of Vegetable oils and fats'
Milk and dairy	01141, 01142, 01143, 01144, 01145, 01146, 01151	'Raw milk', 'Dairy products'
Fish and seafood	01131, 01132, 01133, 01134, 01135, 01136	'Fish and other fishing products; services incidental of fishing (05)', 'Fish products'
Beef	01121	'Cattle', 'Products of meat cattle'
Pork	01122	'Pigs', 'Products of meat pigs'
Poultry	01124	'Poultry', 'Products of meat poultry'
Other meat/animal products	01123, 01125, 01126, 01127, 01128, 01147, 01155	'Meat animals nec', 'Animal products nec', 'Meat products nec'
NEC incl. sugar	01181, 01182, 01183, 01184, 01185, 01186, 01191, 01192, 01193, 01194, 01199	'Sugar cane, sugar beet', 'Sugar', 'Food products nec'

Supplementary Table 3: **Stressors/Impacts from EXIOBASE (v3.8.2)**. The table shows EXIOBASE environmental extensions included in the analysis, as well as their corresponding rows in the stressor (str) and impact (imp) matrices. Stressors are characterized according to their attributed subtype and aggregated for final output according to their type.

**Stressor**	**Type**	**Subtype**	**Row**
Water Consumption Blue - Total			33 (imp)
Phosphorous			105 (imp)
CH4 - combustion - air	GHG emissions	CH4	25 (str)
CH4 - non combustion - Extraction/production of (natural) gas - air	GHG emissions	CH4	68 (str)
CH4 - non combustion - Extraction/production of crude oil - air	GHG emissions	CH4	69 (str)
CH4 - non combustion - Mining of antracite - air	GHG emissions	CH4	70 (str)
CH4 - non combustion - Mining of bituminous coal - air	GHG emissions	CH4	71 (str)
CH4 - non combustion - Mining of coking coal - air	GHG emissions	CH4	72 (str)
CH4 - non combustion - Mining of lignite (brown coal) - air	GHG emissions	CH4	73 (str)
CH4 - non combustion - Mining of sub-bituminous coal - air	GHG emissions	CH4	74 (str)
CH4 - non combustion - Oil refinery - air	GHG emissions	CH4	75 (str)
CH4 - agriculture - air	GHG emissions	CH4	427 (str)
CH4 - waste - air	GHG emissions	CH4	436 (str)
CO2 - combustion - air	GHG emissions	CO2	24 (str)
CO2 - non combustion - Cement production - air	GHG emissions	CO2	93 (str)
CO2 - non combustion - Lime production - air	GHG emissions	CO2	94 (str)
CO2 - agriculture - peat decay - air	GHG emissions	CO2	428 (str)
CO2 - waste - fossil - air	GHG emissions	CO2	439 (str)
HFC - air	GHG emissions	HFC	425 (str)
N2O - combustion - air	GHG emissions	N2O	26 (str)
N2O - agriculture - air	GHG emissions	N2O	430 (str)
PFC - air	GHG emissions	PFC	426 (str)
SF6 - air	GHG emissions	SF6	424 (str)
Cropland - Cereal grains nec	Land use	annual crops	447 (str)
Cropland - Crops nec	Land use	annual crops	448 (str)
Cropland - Fodder crops-Cattle	Land use	annual crops	449 (str)
Cropland - Fodder crops-Meat animals nec	Land use	annual crops	450 (str)
Cropland - Fodder crops-Pigs	Land use	annual crops	451 (str)
Cropland - Fodder crops-Poultry	Land use	annual crops	452 (str)
Cropland - Fodder crops-Raw milk	Land use	annual crops	453 (str)
Cropland - Oil seeds	Land use	annual crops	454 (str)
Cropland - Paddy rice	Land use	annual crops	455 (str)
Cropland - Plant-based fibers	Land use	annual crops	456 (str)
Cropland - Sugar cane, sugar beet	Land use	annual crops	457 (str)
Cropland - Wheat	Land use	annual crops	459 (str)
Other land Use: Total	Land use	extensive forestry	461 (str)
Forest area - Marginal use	Land use	extensive forestry	466 (str)
Forest area - Forestry	Land use	intensive forestry	460 (str)
Permanent pastures - Grazing-Cattle	Land use	pasture	462 (str)
Permanent pastures - Grazing-Meat animals nec	Land use	pasture	463 (str)
Permanent pastures - Grazing-Raw milk	Land use	pasture	464 (str)

Supplementary Table 3: **Stressors/Impacts from EXIOBASE (v3.8.2)** ***(continued).***

**Stressor**	**Type**	**Subtype**	**Row**
Cropland - Vegetables, fruit, nuts	Land use	permanent crops	458 (str)
Infrastructure land	Land use	urban	465 (str)
N - agriculture - water	Nitrogen	N	429 (str)
N - waste - water	Nitrogen	N	441 (str)
NH3 - combustion - air	Nitrogen	NH3	29 (str)
NH3 - agriculture - air	Nitrogen	NH3	431 (str)
NH3 - waste - air	Nitrogen	NH3	442 (str)
NOx - combustion - air	Nitrogen	NOx	28 (str)
NOX - agriculture - air	Nitrogen	NOx	432 (str)
NOX - waste - air	Nitrogen	NOx	443 (str)

Supplementary Table 4: **Sample characteristics by country.** Sample characteristics based on Eurostat Household Budget Survey (HBS) 2010 and 2015, Konsumerhebung (KE) 2014/2015, and Einkommens- und Verbrauchsstichprobe (EVS) 2018. All values are rounded means weighted using sampling weights. Household size is computed according to the modified OECD scale, which assigns a value of 1 to the household head, of 0.5 to each additional adult member and of 0.3 to each child. The column "Share of imputed items" displays the share of food item quantities that were imputed through cross-country matching for cases where certain food items were not recorded in a given country's survey (Case 1 matching described in Methods – Demand system estimation - Data).

Country	Data source	Household size (mod. OECD)	Households with children (share)	Urban households (share)	Household head is female (share)	Household head aged 45 or older (share)	Median household income	Sample size (final)	Share of imputed items
**Austria**	KE14	1.55	21.8%	32.6%	35.2%	66.2%	34563	7155	100 %
**Belgium**	HBS2015	1.58	27.1%	60.7%	41.1%	64%	37802	6135	0 %
**Bulgaria**	HBS2010	1.69	19.4%	43.4%	36.8%	83.8%	4650	2976	2 %
**Croatia**	HBS2015	1.86	28.2%	30%	37%	75.9%	12353	2028	0 %
**Cyprus**	HBS2015	1.77	29.2%	53%	38.1%	61.8%	27924	2861	100 %
**Czechia**	HBS2015	1.54	24.1%	33.6%	49.6%	68.5%	12098	2929	35 %
**Denmark**	HBS2015	1.88	23.3%	31.8%	43.1%	63.2%	48306	2203	100 %
**Estonia**	HBS2015	1.55	25.5%	47.4%	52.2%	62.7%	12143	3369	0 %
**Finland**	HBS2015	1.85	19.1%	38.3%	41.6%	64.8%	38000	3642	4 %
**France**	HBS2010	1.55	27.1%	48.1%	39%	62.7%	32851	14884	4 %
**Germany**	EVS	1.84	18.1%	48.1%	42.3%	65.3%	35454	10345	0 %
**Greece**	HBS2015	1.72	24.8%	42.7%	30.1%	71.7%	16675	6150	0 %
**Hungary**	HBS2015	1.60	21.9%	34.7%	45.9%	67.5%	9108	7051	16 %
**Ireland**	HBS2010	1.80	35.8%	37.1%	46.4%	52.4%	39441	4863	0 %
**Italy**	HBS2015	1.61	23.1%	34.7%	33.7%	73.5%	NA	14916	100 %
**Latvia**	HBS2015	1.62	25.7%	51.7%	51.8%	65.5%	8242	3843	2 %
**Lithuania**	HBS2010	1.74	29.7%	46.5%	43.9%	59.7%	9576	6090	0 %
**Luxembourg**	HBS2015	1.63	26.7%	40.2%	30.5%	59.5%	65904	3161	0 %
**Malta**	HBS2015	1.72	28%	92.6%	35.6%	71.1%	24764	3685	100 %
**Netherlands**	HBS2015	1.53	22.4%	58.4%	34.6%	64.8%	32500	14276	100 %
**Poland**	HBS2015	1.80	34%	39.7%	39.6%	62.7%	10790	37145	4 %
**Portugal**	HBS2015	1.70	30%	45.4%	41.8%	69.1%	19610	11238	0 %
**Romania**	HBS2015	1.76	28.6%	34%	31.8%	62.7%	6468	29865	13 %
**Slovakia**	HBS2015	1.88	30.8%	38.1%	34.8%	56.9%	15376	4785	0 %
**Slovenia**	HBS2015	1.72	26.4%	20.5%	51.7%	65.9%	22375	3749	16 %
**Spain**	HBS2015	1.69	25.4%	51.5%	33.7%	67.5%	24882	21989	24 %
**Sweden**	HBS2015	1.50	23.2%	26%	38.7%	59.5%	35633	2802	100 %

Supplementary Table 5: Symbols used in section *Methods – Demand system estimation.* The table shows the definitions of symbols used in section *Methods – Demand system estimation* ordered by first mention in the text.

Parameter	Definition
$\mathbf{z}_h$	observable preference-related characteristics of household h (L-vector)
$\boldsymbol{\varepsilon}_h$	unobservable preference-related characteristics of household h (n-vector)
x_h	log nominal total food expenditures (scalar)
C	log expenditure function (or cost function)
$\mathbf{p}_h$	log normalized prices encountered by household h (n-vector)
u_h	utility level of household h (scalar)
$\mathbf{w}_h$	observed expenditure shares of household h (n-vector)
$\boldsymbol{\omega}(\mathbf{p}_h, u_h, \mathbf{z}_h, \boldsymbol{\varepsilon}_h)$	Hicksian budget share function
V	indirect utility function
y_h	implicit utility of household h, interpretable as a measure of log real expenditure (scalar)
g	implicit utility function
$\boldsymbol{\omega}(\mathbf{p}_h, y_h, \mathbf{z}_h, \boldsymbol{\varepsilon}_h)$	implicit Marshallian budget share function
$\bar{\mathbf{w}}$	sample mean expenditure shares (n-vector)
$\bar{y}$	sample median implicit utility of household (scalar)
$\mathbf{b}_r$	coefficients (n-vector, for each r)
A	coefficient matrix ($n \times n$-matrix)
B	coefficient matrix ($n \times n$-matrix)
C	coefficient matrix ($L \times n$-matrix)
D	coefficient matrix ($L \times n$-matrix)
$\boldsymbol{\epsilon_h}$	error term (unobserved preference heterogeneity)(n-vector)
E	coefficient matrix ($L \times n$-matrix)
w^*_{hi}	latent (unobserved) expenditure share of household h for food category i (scalar)
d_{hi}	binary selection indicator (=1 if household h has positive expenditure on food category i) (scalar)
d^*_{hi}	latent (unobserved) variable underlying d_{hi} representing the propensity of household h to spend on food category i (scalar)
s_h	household characteristics (k-vector)
γ_i	coefficients (k-vector)
ζ_{hi}	error term (scalar)
$\Phi(\cdot)$	standard normal cumulative distribution function (cdf)
$\phi(\cdot)$	standard normal probability density function (pdf)
$\hat{\Phi}_h$	identity matrix with the diagonal elements replaced by the estimated cdf values of household h given by eq. (11) ($n \times n$-matrix)
$\hat{\phi}_h$	identity matrix with the diagonal elements replaced by the estimated pdf values of household h given by eq. (11) ($n \times n$-matrix)
$\mathbf{f}$	coefficients (n-vector)
UV_{hi}	unit value (expenditures/quantity) of household h in food category i (scalar)
α_i	intercept term (scalar)
$\mathbf{t_h}$	household characteristics (m-vector)
$\boldsymbol{\beta}_i$	coefficients (m-vector)
ξ_{hi}	error term (scalar)
η^{PE}_h	compensated price elasticities ($n \times n$-matrix)
$\tilde{\omega}_h$	identity matrix with the ones replaced by the budget shares $\mathbf{w}_h$ ($n \times n$ matrix)
η^{EE}_h	compensated expenditure elasticities (n-vector)

Supplementary Table 6: **Social cost estimates.** The table shows the social costs of greenhouse gases [1, 2], phosphorus [3], and nitrogen [4] assumed by spatial unit.

Unit	Stressor	Value (EUR)
Global	CO2	170.2 / 249.6
Global	CH4	1453.5 / 2131.4
Global	N2O	47751.1 / 70018.4
EU	Phosphorus	152.4
Austria	NOx	33.5
Austria	NH3	19.3
Austria	N	30.9
Belgium	NOx	20.2
Belgium	NH3	39.5
Belgium	N	28.9
Bulgaria	NOx	9.6
Bulgaria	NH3	7.8
Bulgaria	N	2.8
Cyprus	NOx	14.4
Cyprus	NH3	12.9
Cyprus	N	20.7
Czechia	NOx	25.8
Czechia	NH3	26.0
Czechia	N	14.7
Denmark	NOx	15.8
Denmark	NH3	11.0
Denmark	N	42.4
Estonia	NOx	4.3
Estonia	NH3	6.0
Estonia	N	2.9
Finland	NOx	6.9
Finland	NH3	4.4
Finland	N	25.7
France	NOx	26.9
France	NH3	16.8
France	N	23.3
Germany	NOx	36.2
Germany	NH3	26.5
Germany	N	24.4
Greece	NOx	2.8
Greece	NH3	4.0
Greece	N	17.6
Hungary	NOx	19.1
Hungary	NH3	14.5
Hungary	N	9.6
Ireland	NOx	12.9
Ireland	NH3	3.5
Ireland	N	29.6
Italy	NOx	20.5
Italy	NH3	15.9
Italy	N	22.2
Latvia	NOx	5.4
Latvia	NH3	5.2
Latvia	N	3.5
Lithuania	NOx	6.6
Lithuania	NH3	2.9
Lithuania	N	2.5
Luxembourg	NOx	33.7
Luxembourg	NH3	35.3
Luxembourg	N	81.2
Malta	NOx	15.3
Malta	NH3	14.6
Malta	N	15.9
Netherlands	NOx	25.5
Netherlands	NH3	30.5
Netherlands	N	32.0
Poland	NOx	14.6
Poland	NH3	13.9

Supplementary Table 6: **Social cost estimates *(continued)*.**

Unit	Stressor	Value (EUR)
Poland	N	6.6
Portugal	NOx	4.5
Portugal	NH3	4.9
Portugal	N	15.1
Romania	NOx	9.8
Romania	NH3	7.9
Romania	N	3.3
Slovakia	NOx	19.7
Slovakia	NH3	19.2
Slovakia	N	12.8
Slovenia	NOx	26.7
Slovenia	NH3	19.9
Slovenia	N	17.0
Spain	NOx	8.7
Spain	NH3	5.6
Spain	N	21.3
Sweden	NOx	10.8
Sweden	NH3	8.0
Sweden	N	40.2

Supplementary Table 7: **Comparison of own-price elasticities across studies.** The table compares own-price elasticities derived in this paper (min, median and max of all EU27 country means) with own-price elasticities derived by five relevant studies conducted in EU27 member states and six meta analyses. The entire set of country-specific mean uncompensated own- and cross-price elasticities, as well as expenditure elasticities, along with their standard deviations computed in this study is provided in the Supplementary Material 3.

Study	Bread and cereals	Fruits and vegetables	Veg. oils and fats	Milk and dairy	Fish and seafood	Beef	Pork	Poultry	Other meat/ animal products	NEC incl. sugar
**Studies in EU27 countries**										
Bonnet et al. (2018)[5] - France	-	-	-	Dairy products: -0.58	Fish, shellfish: -1.24	Beef: -1.34	Pork: -1.12	Chicken: -1.45<br>Other poultry: -1.41	Other meat: -1.53<br>Eggs: -1.24	Ready-made meals: -1.27
Dogbe & Gil (2018)[6] - Spain (Catalonia)	Grains & grain-based products: -0.29	Veg. & veg. products: -0.65<br>Starchy roots, tubers, legumes, nuts & oilseeds: -0.61<br>Fruit & fruit products: -0.75	Plant-based fats: -0.38	Cheese: -0.26<br>Milk & dairy products: -0.64	Fish & seafood: -0.40	Beef, veal & lamb: -0.16	Pork: -0.80	Poultry, eggs & other fresh meat: -0.85	Poultry, eggs, & other fresh meat: -0.85<br>Processed meat products: -0.34	Sugar, confectionery & prepared desserts: -0.58<br>Composite dishes: -0.47<br>Snacks & other foods: -0.67
Edjabou & Smed (2013)[7] - Denmark	Flour & Bread: -0.95<br>Rice & Pasta: -0.56	Fresh vegetables: -0.85<br>Frozen vegetables: -1.15<br>Fruit: -1.09	Margarine: -1.03	Milk: -0.48<br>Cheese: -1.21<br>Curdled milk: -0.98<br>Other dairy: -1.66<br>Butter: -1.08	Fish: -0.79<br>Canned Fish: -0.99	Beef: -1.18	Pork: -1.18	Poultry: -1.44	Eggs: -1.42<br>Other meat: -1.00<br>Sliced meat: -1.03	Sugar: -0.93<br>Biscuits & cakes: -1.03<br>Other foods: -1.08
Garcia-Muros et al. (2017)[8] - Spain	Cereals: -0.84	Fruits: -1.19<br>Vegetables: -1.13<br>Potatoes: -0.37	Oils: -0.22	Milk: -0.30<br>Dairy: -0.57	Fish: -0.58	Beef: -1.31	Pork: -0.74	Poultry: -0.68	Eggs: -0.74	Sugar: -0.99
Roosen et al. (2022)[9] - Germany	-	-	-	-	-	Beef & veal: -0.96	Pork: -0.97	Poultry: -0.86	Mixtures: -0.95	-
**Meta Analyses**										
Andreyeva et al. (2010)[10] - US	Cereals: -0.60	Fruit: -0.70<br>Vegetables: -0.58	Fats/oils: -0.48	Dairy: -0.65<br>Milk: -0.59<br>Cheese: -0.44	Fish: -0.50	Beef: -0.75	Pork: -0.72	Poultry: -0.68	Eggs: -0.27	Sweets/sugars: -0.34
Femenia (2019)[11] - EU	Cereals: -0.19	Fruits & vegetables: -0.49	Oils & Fat: -0.17	Dairy: -0.55	-	-	-	-	Meat: -0.49	Other Food: -0.53
Gallet (2010)[12] - global	-	-	-	-	Fish: -1.167	Beef: -0.99	Pork: -0.91	Poultry: -0.78	Lamb: -1.06<br>Meat: -0.85	-
Green et al (2013)[13] - high income countries	Cereals: -0.43	Fruit & vegetables: -0.53	Fats & oils: -0.42	Dairy: -0.6	Fish: -0.61	Meat: -0.6<br>Eggs: -0.36				Sweets, confectionery & sweetened beverages: -0.56<br>Other: -0.77
Bouyssou et al (2024)[14] - EU	Grains: -0.99	Fruits & vegetables, pulses, & tubers: -0.85	Fats: -0.83	Dairy: -0.08 – -2.16<br>Cheese: -1.22	Seafood: -1.03	Beef: -0.75	Pork: -0.79	Poultry: -0.84	Other meat: -0.44<br>Eggs: -0.97	Other food: -1.06
Cornelsen et al (2014)[15] - high income countries	Cereals: -0.43	Fruit and vegetables: -0.53	Fats & oils: -0.42	Dairy: -0.6	Fish: 0.61	Meat: 0.6				Sweets: -0.56
**This paper: EU27 countries...**										
min	-1.11	-1.12	-1.30	-1.04	-1.45	-2.03	-1.78	-1.90	-1.07	-1.65
median	-0.58	-0.91	-0.65	-0.88	-0.83	-1.03	-0.89	-0.77	-0.60	-0.77
max	-0.20	-0.62	-0.23	-0.65	-0.03	-0.23	-0.24	-0.20	0.26	0.29

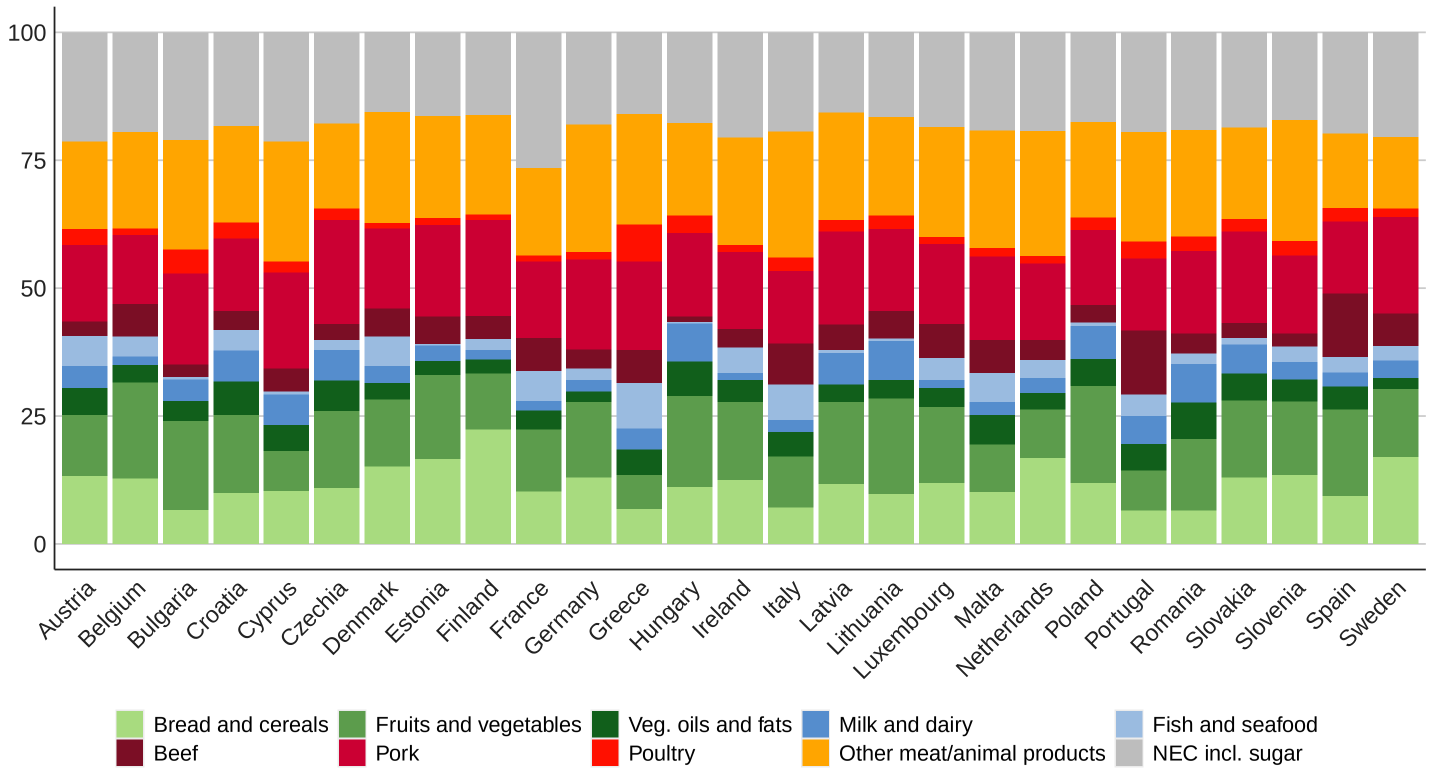

Supplementary Fig. 1: **Budget shares of different food categories by country based on representative household expenditure surveys.** Data sources for each country are listed in Supplementary Table 4. Values are weighted by sample weights to ensure representativeness. NEC denotes not elsewhere classified food items.

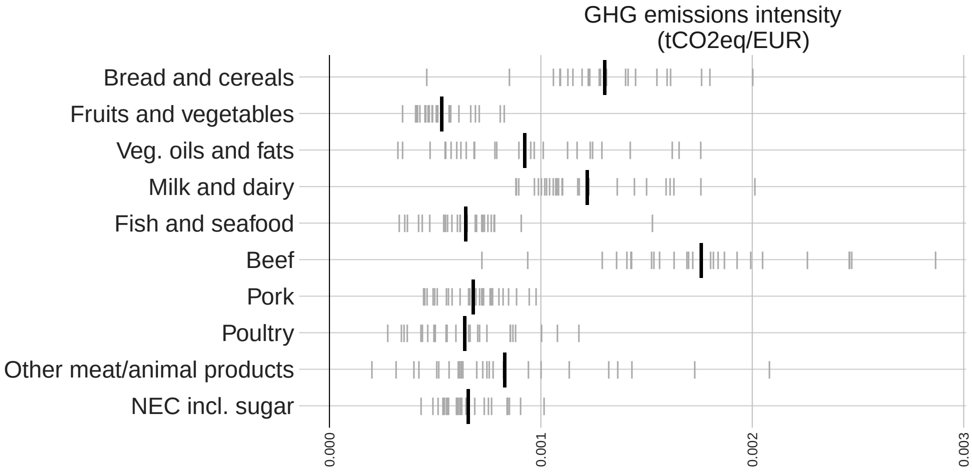

Supplementary Fig. 2: **Greenhouse gas emission intensities of demand by food category**. Intensities are based on EXIOBASE for the year 2019 in tCO_2eq/EUR (own computation). Grey bars indicate country-specific demand intensities; black bars indicate the category-specific (unweighted) mean demand intensity across all EU27 countries. Note that the intensities are expressed as emissions per monetary unit and thus depend on emissions per physical unit as well as economic value per physical unit. For example, the category Bread and cereals may have a relatively high emission intensity, not because of high emissions per unit of weight, but due to lower prices per unit of weight.

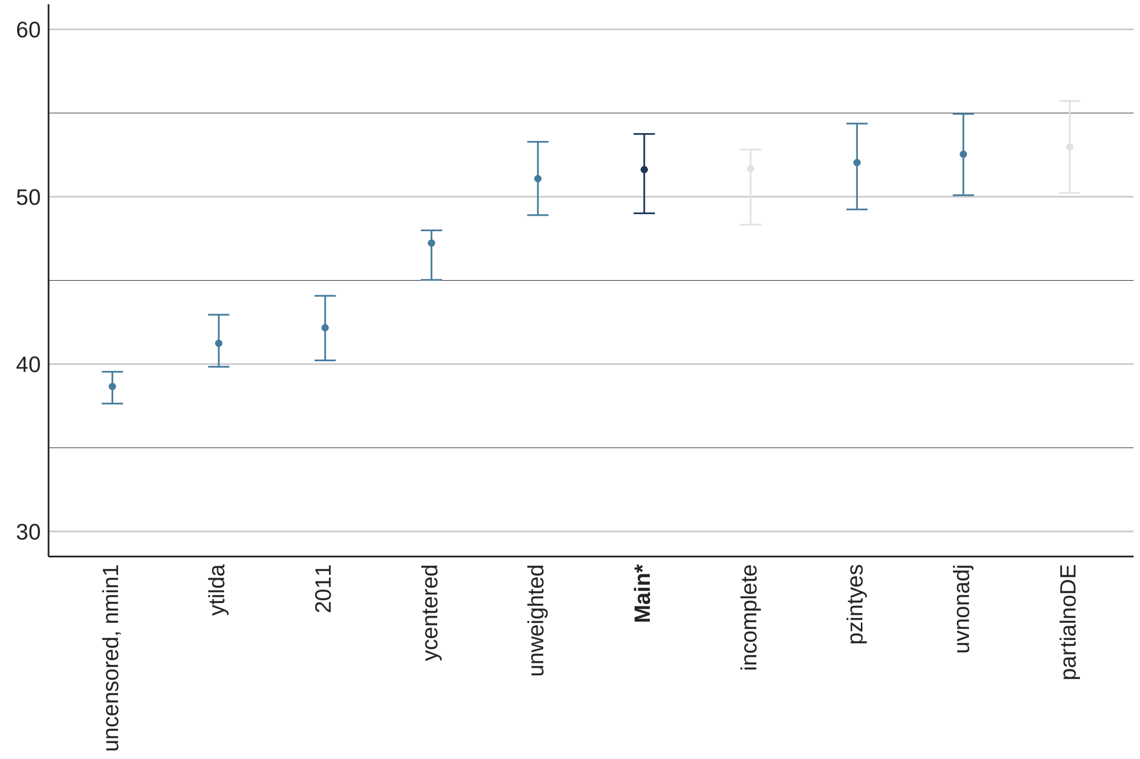

Supplementary Fig. 3: **Robustness of GHG emission price estimates in euros across different demand system and EXIOBASE base year specifications.** The main specification is compared with specification (1) *uncensored, nmin1* (ignores censored distribution of the dependent variable of the demand system and uses only $n-1$ equations recovering the residual food category through the adding up-restriction), specification (2) *ytilda* (uses mean budget share $\bar{w}$ to construct the Stone index $\tilde{y}$ as opposed to the household-specific budget share w used to construct y), specification (3) *2011* (based on EXIOBASE year 2011 using real data points only), specification (4) *ycentered* (uses a median-centered y instead of the absolute value), specification (5) *unweighted* (uses no household weights in the SUR estimation), specification (6) *incomplete* (uses numéraire good to avoid the weak separability assumption, results for 26 countries excl. Germany), specification (7) *pzintyes* (accounting for interactions between household characteristics and prices), specification (8) *uvnonadj* (uses unit values not adjusted for quality choices of the household), and specification (9) *partialnoDE* (main specification excl. Germany, only for comparison with specification *incomplete*). Uncertainty bars represent the range between the minimum and maximum values of the $b = 100$ computed GHG emission price levels based on the $b = 100$ bootstrapped elasticity estimates for each specification.

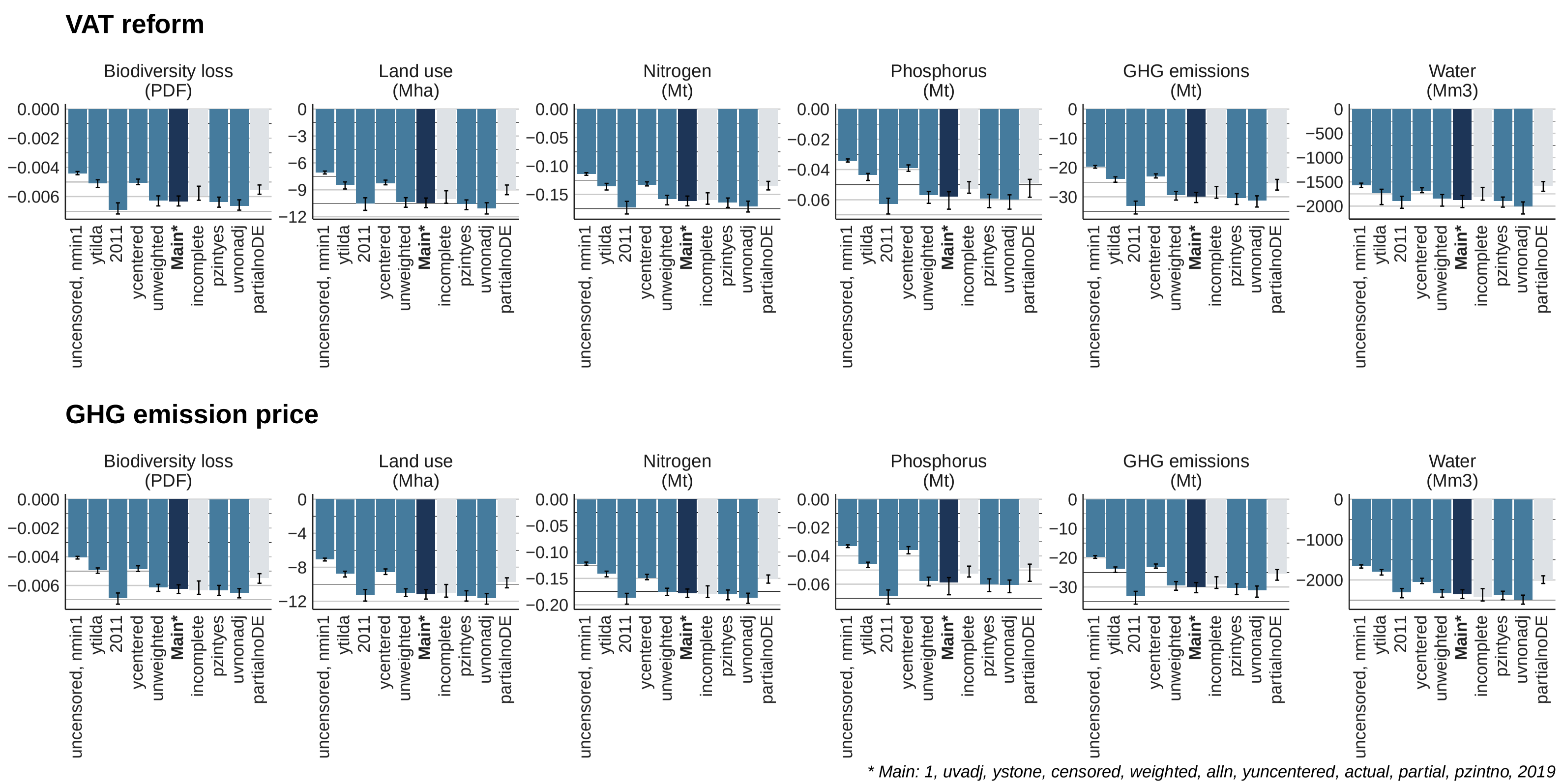

Supplementary Fig. 4: **Robustness of environmental footprint reduction estimates across different demand system and EXIOBASE base year specifications.** The main specification is compared with specification (1) *uncensored, nmin1* (ignores censored distribution of the dependent variable of the demand system and uses only $n-1$ equations recovering the residual food category through the adding up-restriction), specification (2) *ytilda* (uses mean budget share $\bar{w}$ to construct the Stone index $\tilde{y}$ as opposed to the household-specific budget share w used to construct y), specification (3) *2011* (based on EXIOBASE year 2011 using real data points only), specification (4) *ycentered* (uses a median-centered y instead of the absolute value), specification (5) *unweighted* (uses no household weights in the SUR estimation), specification (6) *incomplete* (uses numéraire good to avoid weak separability assumption, results for 26 countries excl. Germany), specification (7) *pzintyes* (accounting for interactions between household characteristics and prices), specification (8) *uvnonadj* (uses unit values not adjusted for quality choices of the household), and specification (9) *partialnoDE* (main specification excl. Germany, only for comparison with specification *incomplete*). The underlying GHG emission price varies by specification (see Fig. 3). Uncertainty bars represent the range between the minimum and maximum values of the $b = 100$ computed footprint reductions based on the $b = 100$ bootstrapped elasticity estimates for each specification.

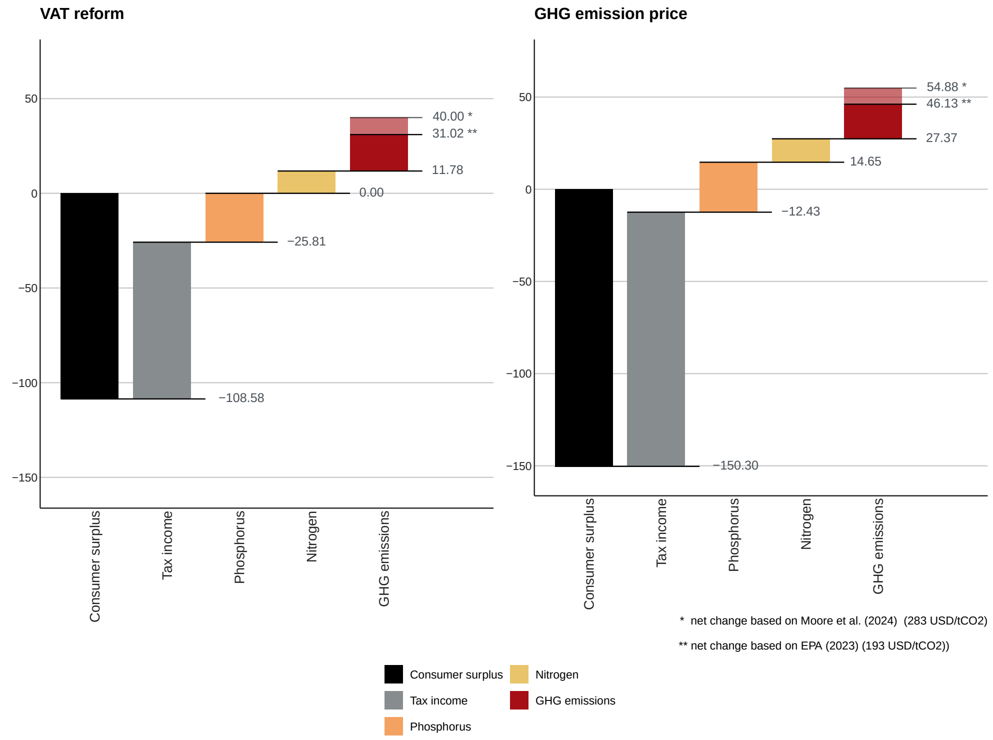

Supplementary Fig. 5: **Policy-induced monetised social welfare change per household.** Reduction in consumer surplus, increase in tax revenue, and mean monetised changes in environmental footprints resulting from the removal of value-added tax reductions for meat products (*VAT reform*) and the implementation of a GHG emission price of 51.63 EUR/tCO_2eq on all food products (*GHG emission price*), measured in euros per household across all EU27 households. Global GHG emission benefits are valued based on the global social costs (SC) of greenhouse gases (CO_2, CH_4 and N_2O) reported in [1] and the mean global social cost of carbon estimated by [2]. As [2] do not provide social cost estimates for CH_4 and N_2O, those values are imputed using the reported SCC of 283 USD/tCO_2 which is then scaled using the ratios between SC-CO_2 and SC-CH_4 and SC-N_2O taken from [1]. Local phosphorus and nitrogen (N, NH_3 and NO_x) emission benefits are valued based on domestic social costs [3, 4]. Demand reductions are assumed to be proportional to current trade and consumption patterns. Total environmental benefits represent lower bound estimates as changes in biodiversity loss, land use and water consumption are not monetised due to a lack of social cost estimates.

References

[1] EPA. Report on the Social Cost of Greenhouse Gases: Estimates incorporating recent scientific advances (2023).

[2] Moore, F. C. *et al.* Synthesis of evidence yields high social cost of carbon due to structural model variation and uncertainties. *Proceedings of the National Academy of Sciences* **121**, e2410733121 (2024).

[3] Matthey, A. & Bünger, B. Methodenkonvention 3.1 zur Ermittlung von Umweltkosten - Kostensätze (2020).

[4] van Grinsven, H. J. M. *et al.* Reducing external costs of nitrogen pollution by relocation of pig production between regions in the european union. *Regional Environmental Change* **18**, 2403–2415 (2018).

[5] Bonnet, C., Bouamra-Mechemache, Z. & Corre, T. An environmental tax towards more sustainable food: Empirical evidence of the consumption of animal products in france. *Ecological Economics* **147**, 48–61 (2018).

[6] Dogbe, W. & Gil, J. M. Effectiveness of a carbon tax to promote a climate-friendly food consumption. *Food Policy* **79**, 235–246 (2018).

[7] Edjabou, L. D. & Smed, S. The effect of using consumption taxes on foods to promote climate friendly diets – the case of denmark. *Food policy* **39**, 84–96 (2013).

[8] García-Muros, X., Markandya, A., Romero-Jordán, D. & González-Eguino, M. The distributional effects of carbon-based food taxes. *Journal of Cleaner Production* **140**, 996–1006 (2017).

[9] Roosen, J., Staudigel, M. & Rahbauer, S. Demand elasticities for fresh meat and welfare effects of meat taxes in germany. *Food Policy* **106**, 102194 (2022).

[10] Andreyeva, T., Long, M. W. & Brownell, K. D. The impact of food prices on consumption: A systematic review of research on the price elasticity of demand for food. *American journal of public health* **100**, 216–222 (2010).

[11] Femenia, F. A meta-analysis of the price and income elasticities of food demand **68**, 78 p. (2019). URL https://hal.science/hal-02103880. 19-03.

[12] Gallet, C. A. Meat meets meta: A quantitative review of the price elasticity of meat. *American Journal of Agricultural Economics* **92**, 258–272 (2010).

[13] Green, R. *et al.* The effect of rising food prices on food consumption: systematic review with meta-regression. *BMJ* **346**, f3703–f3703 (2013).

[14] Bouyssou, C. G., Jensen, J. D. & Yu, W. Food for thought: A meta-analysis of animal food demand elasticities across world regions. *Food Policy* **122**, 102581 (2024).

[15] Cornelsen, L. *et al.* What happens to patterns of food consumption when food prices change? evidence from a systematic review and meta-analysis of food price elasticities globally. *Health economics* **24**, 1548–1559 (2015).
