# Supplementary material for: Environmental impacts from European food consumption can be reduced with carbon pricing or a value-added tax reform
Source: Nat Food. 2026 Jan 20;7(1):74–87. doi: 10.1038/s43016-025-01284-y (PMC12851928; doi:10.1038/s43016-025-01284-y)
Supplement: Supplementary file 2 — Reporting Summary [file 43016_2025_1284_MOESM2_ESM.pdf]

nature portfolio

Corresponding author(s): Charlotte Plinke

Last updated by author(s): Dec 2, 2025

Reporting Summary

Nature Portfolio wishes to improve the reproducibility of the work that we publish. This form provides structure for consistency and transparency in reporting. For further information on Nature Portfolio policies, see our Editorial Policies and the Editorial Policy Checklist.

Statistics

For all statistical analyses, confirm that the following items are present in the figure legend, table legend, main text, or Methods section.

n/a	Confirmed	
☐	☒	The exact sample size (n) for each experimental group/condition, given as a discrete number and unit of measurement
☒	☐	A statement on whether measurements were taken from distinct samples or whether the same sample was measured repeatedly
☒	☐	The statistical test(s) used AND whether they are one- or two-sided<br>*Only common tests should be described solely by name; describe more complex techniques in the Methods section.*
☒	☐	A description of all covariates tested
☐	☒	A description of any assumptions or corrections, such as tests of normality and adjustment for multiple comparisons
☐	☒	A full description of the statistical parameters including central tendency (e.g. means) or other basic estimates (e.g. regression coefficient) AND variation (e.g. standard deviation) or associated estimates of uncertainty (e.g. confidence intervals)
☒	☐	For null hypothesis testing, the test statistic (e.g. F, t, r) with confidence intervals, effect sizes, degrees of freedom and P value noted<br>*Give P values as exact values whenever suitable.*
☒	☐	For Bayesian analysis, information on the choice of priors and Markov chain Monte Carlo settings
☒	☐	For hierarchical and complex designs, identification of the appropriate level for tests and full reporting of outcomes
☒	☐	Estimates of effect sizes (e.g. Cohen's d, Pearson's r), indicating how they were calculated

Our web collection on statistics for biologists contains articles on many of the points above.

Software and code

Policy information about availability of computer code

Data collection	This study did not collect new data. Code for processing EXIOBASE 3.8.2. was partly retrieved from Bruckner et al. (2023).
Data analysis	We used R version 4.3.1 for data processing, policy simulation and the creation of display items. We used Stata (15 and 18) to estimate the demand system. The code is publicly available on Zenodo (https://doi.org/10.5281/zenodo.17295051).

For manuscripts utilizing custom algorithms or software that are central to the research but not yet described in published literature, software must be made available to editors and reviewers. We strongly encourage code deposition in a community repository (e.g. GitHub). See the Nature Portfolio guidelines for submitting code & software for further information.

Data

Policy information about availability of data

All manuscripts must include a data availability statement. This statement should provide the following information, where applicable:

- Accession codes, unique identifiers, or web links for publicly available datasets
- A description of any restrictions on data availability
- For clinical datasets or third party data, please ensure that the statement adheres to our policy

Household microdata were sourced from multiple surveys including Eurostat’s Household Budget Survey (HBS 2010 and 2015, https://ec.europa.eu/eurostat/web/microdata/household-budget-survey), the Konsumerhebung 2014/15 provided by Statistics Austria (https://www.statistik.at/ueber-uns/erhebungen/personen-und-haushaltserhebungen/konsumerhebung) and the Einkommens- und Verbrauchsstichprobe (EVS) 2018 provided by the German Federal Statistical Office (https://

www.forschungsdatenzentrum.de/de/10-21242-63231-2018-00-00-3-1-0). Access to data for research purposes must be requested directly from the relevant statistical agencies. Additional statistics on population size and the number and average size of households by country were retrieved from Eurostat's Population and Demography database (https://ec.europa.eu/eurostat/web/population-demography/demography-population-stock-balance/database) and the Eurostat Labour Force Survey (LFS) database (https://ec.europa.eu/eurostat/web/lfs/database). Environmentally extended multi-regional input-output (MRIO) data were retrieved from EXIOBASE (version 3.8.2) (https://zenodo.org/records/5589597). Land use-related biodiversity loss coefficients were sourced from Bruckner et al. (2023) based on Koslowski et al. (2020). Social costs of greenhouse gases, nitrogen and phosphorus were taken from EPA (2023), Moore et al. (2024), Matthey & Bünger (2020) and van Grinsven et al. (2018). Shapefiles used to create maps are based on Natural Earth Data (https://www.naturalearthdata.com/), were sourced using the rnaturalearth R package (https://cran.r-project.org/web/packages/rnaturalearth/) and in part processed following Bruckner et al. (2023).

Research involving human participants, their data, or biological material

Policy information about studies with human participants or human data. See also policy information about sex, gender (identity/presentation), and sexual orientation and race, ethnicity and racism.

Reporting on sex and gender	Data is analysed at the household level. We use information on whether the household head is identified as female as provided by the Eurostat Household Budget Survey (HBS), the Konsumerhebung 2014/15 provided by Statistics Austria and the Einkommens- und Verbrauchsstichprobe (EVS) 2018 provided by the German Federal Statistical Office.
Reporting on race, ethnicity, or other socially relevant groupings	No information regarding race or ethnicity was collected, analyzed, or reported in this study.
Population characteristics	See above.
Recruitment	We use existing household-level data collected by the national statistical offices of the EU27 member countries. Sampling was conducted by the national statistical offices of the EU27 member countries. We use harmonized data for 25 of 27 countries as provided by Eurostat (https://ec.europa.eu/eurostat/web/microdata/household-budget-survey). For all countries, household weights are provided for inference on the reference population.
Ethics oversight	We requested household data from Eurostat (Household Budget Survey), Statistics Austria (Konsumerhebung 2014/15) and the German Federal Statistical Office (Einkommens- und Verbrauchsstichprobe 2018). The statistical agencies approved our research proposal before granting access to the data.

Note that full information on the approval of the study protocol must also be provided in the manuscript.

Field-specific reporting

Please select the one below that is the best fit for your research. If you are not sure, read the appropriate sections before making your selection.

☐ Life sciences ☒ Behavioural & social sciences ☐ Ecological, evolutionary & environmental sciences

For a reference copy of the document with all sections, see nature.com/documents/nr-reporting-summary-flat.pdf

Behavioural & social sciences study design

All studies must disclose on these points even when the disclosure is negative.

Study description	This study uses quantitative micro-level (household) data representative of each of the EU27 countries from three main sources (see below). Demand elasticities are estimated to model the impact of different tax policies on environmental impacts, which are determined using the environmentally-extended multi-regional input-output model EXIOBASE v.3.8.2.
Research sample	We use existing representative household data for all 27 member countries of the European Union. Variables of interest are expenditure and consumption patterns of private households and their members, as well as their sociodemographic characteristics.<br>For Germany, we use the Einkommens- und Verbrauchsstichprobe (EVS) 2018 provided by provided by the German Federal Statistical Office. The survey population includes all private households with a monthly net household income below 18,000 euros. The EVS is a quota sample with voluntary participation. Extrapolation factors are designed to ensure that the extrapolated results regarding selected characteristics match the population data from the microcensus.<br>For Austria, we use the Konsumerhebung 2014/15 provided by Statistics Austria. The Konsumerhebung is a random sample with voluntary participation.<br>For the remaining 25 member countries of the EU27, we use harmonized household data from the EU Household Budget Survey (HBS 2010 and HBS 2015) provided by Eurostat.<br><br>In addition, we investigate six environmental impact dimensions associated with household food consumption in the EU27 member states in 2019, based on the environmentally-extended multi-regional input-output model EXIOBASE v.3.8.2.
Sampling strategy	For Germany, the EVS is a quota sample with extrapolation factors designed to ensure that the extrapolated results regarding selected characteristics match the population data from the microcensus.<br>For Austria, the Konsumerhebung is a random sample with households being randomly selected from the Central Register of Residents (ZMR).<br>Within the EU Household Budget Survey (HBS), various types of sampling frames were used for sample selection. Czech Republic uses quota sampling, while the remaining samples were selected according to a probability sampling scheme. Population registers and

census data were used as sampling frames. Details are available in the Household Budget Survey 2015 Wave EU Quality Report (https://ec.europa.eu/eurostat/documents/54431/1966394/HBS_EU_QualityReport_2015.pdf/72d7e310-c415-7806-93cc-e3bc7a49b596) and the Household Budget Survey 2010 Wave EU Quality Report (https://ec.europa.eu/eurostat/documents/54431/1966394/LC142-15EN_HBS_2010_Quality_Report_ver2+July+2015.pdf/fc3c8aca-c456-49ed-85e4-757d4342015f).

For the environmental impact analysis, we carefully selected 51 stressors and impacts relevant to the six environmental dimensions. The MRIO model EXIOBASE was selected due to its individual representation of the EU27 member countries, and its detailed and globally consistent resolution of 200 distinct sectors. Specifically, we focused on a subset comprising 14 agricultural and ten food processing sectors.
We use data for the year 2019, which relies on "now-casting" of the economic structure and all environmental satellite accounts except for CO2 emissions. Given that end years of real data points are 2018 for non-CO2 emissions and 2011 for all other environmental accounts, we also present results based solely on real data from 2011.

Data collection	No own data collection was conducted. For details on the data collection conducted by national statistics offices please refer to the documentation of the data sets described above.<br>EXIOBASE v.3.8.2. was downloaded from Zenodo (https://zenodo.org/records/5589597). Biodiversity loss coefficients were retrieved from Bruckner et al. (2023), based on Koslowski et al. (2020). Shapefiles used to create maps are based on Natural Earth ((https://www.naturalearthdata.com/) and the eurostat R package (https://cran.r-project.org/web/packages/eurostat/index.html) and were processed following Bruckner et al. (2023).
Timing	No own data collection was conducted. For details on the timing of the household data collection conducted by national statistics offices please refer to the documentation of the data sets described above.<br>The main analysis uses environmentally-extended input-output data for the year 2019, with additional results based solely on real data from 2011.
Data exclusions	We do not exclude any valid observations. We identify and remove implausible observations if one of the following applies: (1) The household records zero food expenditures, (2) the household's food expenditure constitutes more than 75% of total expenditures, (3) the household reports negative expenditures, (4) the household reports zero expenditures in combination with positive quantities in a given category.<br>No data exclusions were conducted for the environmental impact analysis based on EXIOBASE v.3.8.2.
Non-participation	The response rate of the national household surveys at an EU level lies between 16.5 percent in the Netherlands and 80.0 percent in Romania. In the case of quota sampling (Germany, Czech Republic), the non-response rate is generally unknown. Country-specific response rates are available in the Household Budget Survey 2015 Wave EU Quality Report (https://ec.europa.eu/eurostat/documents/54431/1966394/HBS_EU_QualityReport_2015.pdf/72d7e310-c415-7806-93cc-e3bc7a49b596) and the Household Budget Survey 2010 Wave EU Quality Report (https://ec.europa.eu/eurostat/documents/54431/1966394/LC142-15EN_HBS_2010_Quality_Report_ver2+July+2015.pdf/fc3c8aca-c456-49ed-85e4-757d4342015f).
Randomization	We did not introduce any randomization.

Reporting for specific materials, systems and methods

We require information from authors about some types of materials, experimental systems and methods used in many studies. Here, indicate whether each material, system or method listed is relevant to your study. If you are not sure if a list item applies to your research, read the appropriate section before selecting a response.

Materials & experimental systems

n/a	Involved in the study	
☒	☐	Antibodies
☒	☐	Eukaryotic cell lines
☒	☐	Palaeontology and archaeology
☒	☐	Animals and other organisms
☒	☐	Clinical data
☒	☐	Dual use research of concern
☒	☐	Plants

Methods

n/a	Involved in the study	
☒	☐	ChIP-seq
☒	☐	Flow cytometry
☒	☐	MRI-based neuroimaging

Plants

Seed stocks	*Report on the source of all seed stocks or other plant material used. If applicable, state the seed stock centre and catalogue number. If plant specimens were collected from the field, describe the collection location, date and sampling procedures.*
Novel plant genotypes	*Describe the methods by which all novel plant genotypes were produced. This includes those generated by transgenic approaches, gene editing, chemical/radiation-based mutagenesis and hybridization. For transgenic lines, describe the transformation method, the number of independent lines analyzed and the generation upon which experiments were performed. For gene-edited lines, describe the editor used, the endogenous sequence targeted for editing, the targeting guide RNA sequence (if applicable) and how the editor was applied.*
Authentication	*Describe any authentication procedures for each seed stock used or novel genotype generated. Describe any experiments used to assess the effect of a mutation and, where applicable, how potential secondary effects (e.g. second site T-DNA insertions, mosiacism, off-target gene editing) were examined.*
